# Technologies for Direct Detection of Covalent Protein–Drug Adducts

**DOI:** 10.3390/ph16040547

**Published:** 2023-04-05

**Authors:** Elma Mons, Robbert Q. Kim, Monique P. C. Mulder

**Affiliations:** 1 Department of Cell and Chemical Biology, Leiden University Medical Center, 2300 RC Leiden, The Netherlands; m.w.e.mons@biology.leidenuniv.nl (E.M.);; 2 Institute of Biology Leiden, Leiden University, 2333 BE Leiden, The Netherlands

**Keywords:** covalent inhibitors, chemical probes, covalent warheads, medicinal chemistry, drug discovery, drug development, target validation, mode of action studies, ABPP, mass spectrometry, protein crystallography, NMR

## Abstract

In the past two decades, drug candidates with a covalent binding mode have gained the interest of medicinal chemists, as several covalent anticancer drugs have successfully reached the clinic. As a covalent binding mode changes the relevant parameters to rank inhibitor potency and investigate structure-activity relationship (SAR), it is important to gather experimental evidence on the existence of a covalent protein–drug adduct. In this work, we review established methods and technologies for the direct detection of a covalent protein–drug adduct, illustrated with examples from (recent) drug development endeavors. These technologies include subjecting covalent drug candidates to mass spectrometric (MS) analysis, protein crystallography, or monitoring intrinsic spectroscopic properties of the ligand upon covalent adduct formation. Alternatively, chemical modification of the covalent ligand is required to detect covalent adducts by NMR analysis or activity-based protein profiling (ABPP). Some techniques are more informative than others and can also elucidate the modified amino acid residue or bond layout. We will discuss the compatibility of these techniques with reversible covalent binding modes and the possibilities to evaluate reversibility or obtain kinetic parameters. Finally, we expand upon current challenges and future applications. Overall, these analytical techniques present an integral part of covalent drug development in this exciting new era of drug discovery.

## 1. Introduction

**Figure A1:**
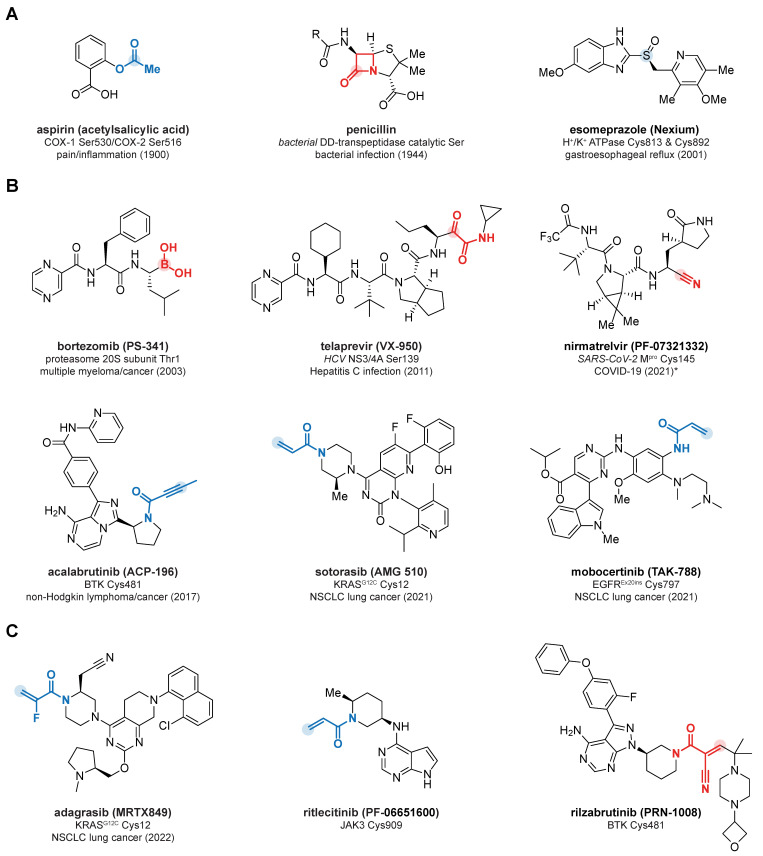
Drugs with a known covalent binding mode. Structure with reversible warheads in red, irreversible warheads in blue, and the covalently modified atom marked. Compound name; protein target and covalently modified amino acid; therapeutic application and year of first clinical approval. (**A**) Approved covalent inhibitors that were later found to have a covalent binding mode. Warhead not indicated for esomeprazole: metabolic activation of the prodrug precedes formation of a disulfide bond with its target [6]. (**B**) Approved targeted covalent inhibitors (TCIs) designed to covalently modify their protein target. (**C**) Covalent inhibitors with a novel protein target and/or warhead.

Among the most prescribed drugs in the US, [1,2] are successful drugs that were later found to have a covalent binding mode (Figure A1A in Appendix A), including established pain killer/anti-inflammatory agent aspirin [3], β-lactam antibiotic penicillin [4], anticoagulant clopidogrel (Plavix) [5], and proton-pump inhibitor (es)omeprazole (Nexium) for gastroesophageal reflux [6]. In the past two decades, the paradigm shift from covalent inhibition as an avoided liability toward the development of targeted covalent inhibitors (TCIs) has led to the approval of various drugs with a covalent binding mode (Figure A1B in Appendix A) [1,7,8]. Covalent targeting of noncatalytic cysteine residues at the ATP-binding site of kinases has since proven to be a successful approach to overcome competition by the native substrate [9,10], as illustrated by clinically approved covalent Bruton’s Tyrosine Kinase (BTK) inhibitors [11,12,13] and covalent (mutant) EGFR inhibitors [14,15,16,17]. Furthermore, a covalent binding mode enabled inhibition of challenging targets for which noncovalent inhibitors could not successfully be developed, as illustrated by two recently approved first-in-class drugs: sotorasib (AMG 510) modifies Cys12 in the oncogenic KRAS^G12C^ mutant [18] and mobocertinib (TAK788) modifies noncatalytic Cys797 of the EGFR^ex20ins^ mutant [19]. An extensive overview of all FDA-approved drugs (1900–2019) with a known covalent mechanism of action that lists their therapeutic application along with the electrophilic warhead has been compiled by De Vita [20]. An update (2020–2022) can be found in the supporting information accompanying this review (Table A1 in Appendix A).

Typically, a covalent adduct is formed when an electrophilic moiety (or warhead) in the inhibitor is positioned in juxtaposition with a nucleophilic residue in the protein target [21,22]. Commonly targeted amino acid residues are catalytic cysteine and serine residues as the activated Cys thiolate and Ser hydroxylate are more nucleophilic (low pKa) than their noncatalytic (protonated) counterparts (Cys: pKa = 8–9, Ser: pKa > 13) [23]. Popular noncatalytic nucleophilic residues include cysteines, lysines, and (*N*-terminal) threonines [23,24]. The selection of a warhead depends on the identity of the amino acid residue, the nucleophilicity of the targeted amino acid residue, and the desired binding mode (reversible or irreversible) [10,24,25]. A warhead should have the right balance between intrinsic chemical reactivity and selectivity, quickly forming a covalent adduct with the desired target but not (or much slower) with undesired cellular components [26,27]. For cysteine-targeting inhibitors, this is typically assessed in indiscriminate thiol reactivity assays with biological thiols such as glutathione (GSH) [28] or cysteine [29]. The acrylamides and related Michael acceptors, employed in several approved kinase inhibitors, are among the most popular warheads for irreversible covalent targeting of noncatalytic cysteine thiols as the balance of their intrinsic chemical reactivity and selectivity results in a favorable safety profile [10,20]. Available warheads, popular as well as upcoming, and their application have been reviewed elsewhere [23,24,30,31]. Generally, the development of novel TCIs entails the introduction of a warhead onto a potent noncovalent scaffold [1,21,32,33,34], or high-throughput screening (HTS) of small molecule covalent ligands [35,36,37] or covalent fragment libraries [31,36,38,39,40,41,42,43], with structure-based lead optimization supported by in silico approaches (e.g., covalent docking, virtual screening [32,44,45,46]).

Reversible covalent inhibition is becoming increasingly popular [47,48,49,50,51,52,53] as it combines the high affinity and long residence time of a covalent binding mode with a reduced risk of undesired idiosyncratic toxicity associated with the intrinsic ability to irreversibly modify off-target proteins [49]. This approach is especially useful for targets with a relatively short cellular half-life as (proteasomal/proteolytic) degradation of the protein target will induce the release of the reversibly bound covalent inhibitor that can engage in the inhibition of another target protein. The introduction of an electron-withdrawing cyano group on the α-position of an irreversible covalent acrylamide warhead generates the cyanoacrylamide warhead, which was found to convert the inhibitors into reversible covalent inhibitors with a tunable residence time [54,55]. The cyanoacrylamide moiety has gained popularity [56,57,58], most notably illustrated by reversible covalent BTK inhibitor rilzabrutinib (PRN-1008) [49], which is currently in phase III clinical trials (Figure A1C in Appendix A). Another recent example of the success of reversible covalent inhibition is nirmatrelvir (PF-07321332), the principle/novel component of Pfizer’s oral antiviral agent Paxlovid that received emergency use authorization in 2021 for the treatment of mild-to-moderate coronavirus disease (COVID-19) [47,59]. Nirmatrelvir inhibits SARS-CoV-2 main protease M^pro^ by formation of a reversible covalent thioimidate bond between an electrophilic nitrile warhead and the catalytic cysteine thiolate [47].

**Figure 1:**
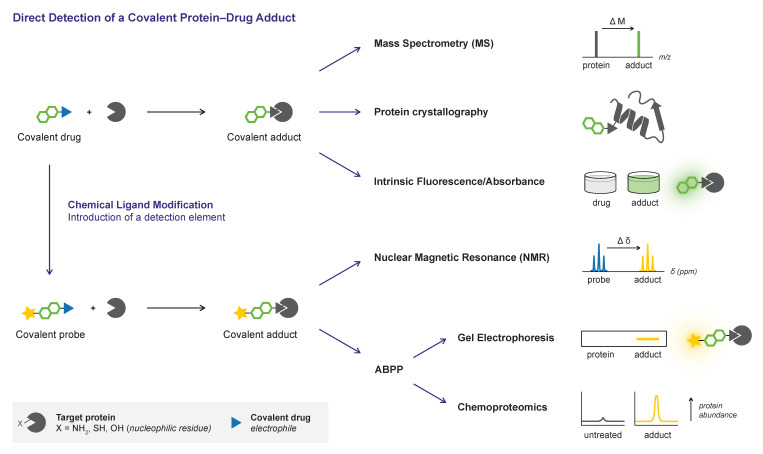
Validation of a covalent binding mode by direct detection of the covalent protein–drug adduct.

As a covalent binding mode changes the relevant parameters to rank drug potency and investigate structure–activity relationship (SAR) [1,60,61,62,63], it is important to gather experimental evidence on the existence of a covalent protein–drug adduct. Compounds sharing a warhead do not necessarily have the same covalent reactivity, and an electrophile is no guarantee for a covalent protein–drug adduct. Most claims pertaining a covalent binding mode are based on data obtained with the drug itself, but there still are examples of clinically approved drugs for which the covalent binding mode is not explicitly demonstrated but assumed based on related compounds or covalent docking (e.g., remdesivir [64]). In this work, we review the available methods in the toolbox to validate covalent adduct formation, rather than identification of novel covalent ligands/inhibitors (Figure 1). Please note that the term inhibitor implies that target binding impairs protein function or blocks a protein–protein interaction, thus not reflecting covalent (partial) agonists [65,66,67] and covalent PROTACs [68,69]. We will use the more appropriate neutral term covalent ligand as it describes any covalent modifier without specifying how target engagement affects protein function/binding. We focus on technologies enabling direct detection of the covalent protein–ligand adduct under conditions that distinguish covalent adducts from noncovalent complexes (e.g., an increase in the total mass under denaturing conditions), while indirect covalent adduct detection protocols (e.g., competitive activity-based probe labeling) are occasionally mentioned to exemplify their use as orthogonal validation tools.

Most direct methods only discriminate between noncovalent and covalent protein modification, while others are more informative and provide direct evidence about which amino acid residue is modified. Although it is generally safe to assume that the most nucleophilic (catalytic) amino acid residue will be targeted for covalent modification, TCIs that were unexpectedly found to covalently modify allosteric (less nucleophilic) residues [70] or even a completely different amino acid [71] illustrate why it is important to identify the modified amino acid residue. Importantly, covalent adduct formation is not completed instantly upon treating the target protein with an excess covalent ligand [60]. Unless otherwise noted, all procedures involve incubation of protein target and covalent ligand for a sufficient time (ranging from minutes to hours) to allow covalent adduct formation prior to analysis, as it is not possible to detect a covalent adduct that has not (yet) been formed. Conversion to covalent adduct does not have to be complete but high amounts of unbound protein can complicate detection, especially if unbound protein cannot easily be removed. The focus of this review is on the qualitative detection of covalent protein–ligand adducts but some of the methods enable quantification of time-dependent covalent occupancy, which might be employed to calculate kinetic rate constants reflecting irreversible covalent inhibitor potency [1,60,61]. Details on the kinetic background of covalent adduct formation and potency are beyond the scope of this work [60,62], but compatibility with quantification of covalent occupancy will be highlighted. Moreover, special attention will be paid to the compatibility with reversible covalent ligands and reversibility assays to assess the (ir)reversible ligand binding mode. Detection of reversible covalent adducts has its unique challenges compared to irreversible covalent adduct detection: detection (and purification) of the reversible covalent protein–ligand adduct [54] is more complicated as the unbound enzyme and covalent adduct are at an equilibrium, and the covalent occupancy is thus driven by the concentration of (excess) inhibitor [48,60]. Furthermore, standard sample preparation conditions (e.g., denaturation, proteolytic digestion, dilution), designed to induce noncovalent inhibitor dissociation, can also induce dissociation of reversible covalent ligands [54,72]. Traditional reversibility assays are based on regained enzymatic activity after rapid dilution [73] or washout [34], or on detection of released unbound inhibitor upon protein denaturation/digestion [55] or chasing with a competitive irreversible ABP [74]. These assays serve to evaluate the reversibility of the adduct formation but irreversible protein modification provides by no means direct evidence of covalency; a covalent drug can have a reversible binding mode, and noncovalent binders can be irreversible [60].

In this work, we will discuss methods for the direct detection of covalent protein–ligand adducts (an overview of the methods can be found in Table 1). In general, whether the covalent adduct will be detected using a certain technique depends on the intrinsic properties of the protein target (e.g., mass, ionizability, crystalline) as well as the inhibitor/ligand (e.g., binding mode, solubility, fluorescence). Each method will be illustrated with examples of advantages and limitations, with specific attention to compatibility with reversible covalent inhibition, identification of the modified amino acid residue, and application in the (kinetic) evaluation of inhibitor binding mode and/or potency.

We start with techniques to detect the covalent protein–drug adduct without chemical modification of the ligand using the same compound stocks prepared for biochemical in vitro/in vivo assays. Predominantly used techniques mass spectrometry (Section 2) and protein crystallography (Section 3) will be discussed first, followed by less ubiquitous detection based on the changes in intrinsic spectroscopic properties of the ligand upon covalent adduct formation (Section 4). Alternatively, ^13^C NMR analysis (Section 5) and activity-based protein profiling (Section 6) require chemical modification of the covalent ligand to enable detection of the covalent protein–drug adduct (e.g., introduction of a bioorthogonal handle, reporter tag, or isotope labeling). Finally, we expand on current challenges and future applications (Section 7).

## 2. Mass Spectrometry (MS)

**Figure 2:**
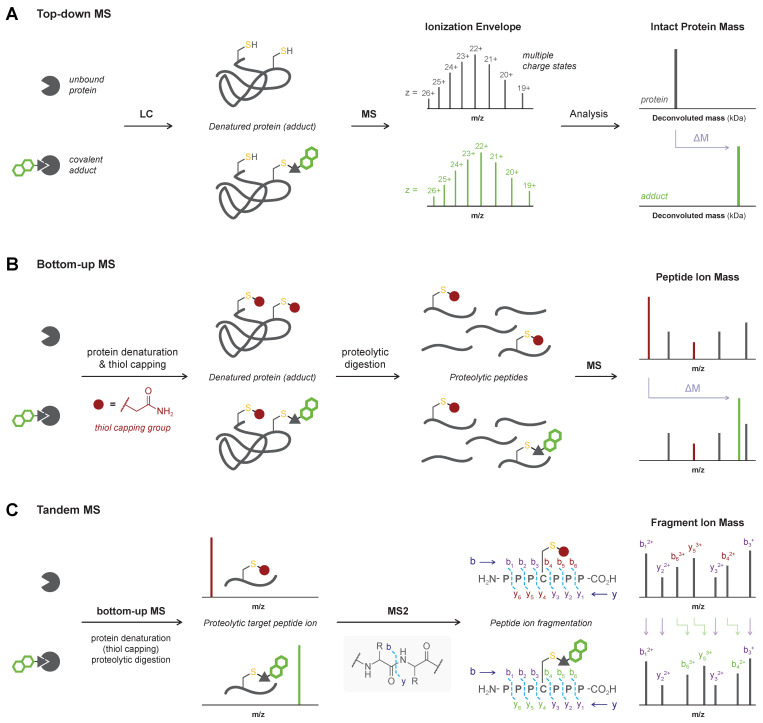
Schematic overview of MS-based methodologies for detection of covalent protein–drug adducts. (**A**) Intact protein analysis by top-down MS analysis. Samples containing unbound protein (top) or covalent adduct (bottom) are resolved by liquid chromatography (LC) to promote ligand dissociation in noncovalent complexes and remove free ligand. Intact protein and covalent adduct are subjected to MS analysis, where they are ionized multiple times (z ≥ 1) generating an ionization envelope originating from the various charge states, from which the deconvoluted total mass is calculated. The covalent protein–drug adduct has a higher deconvoluted mass than the unbound protein. (**B**) Bottom-up MS analysis. Samples containing unbound protein (top) or covalent adduct (bottom) are subjected to proteolytic digestion, with optional capping of free thiols using thiol-reactive reagent iodoacetamide (IAc) before or after digestion, followed by MS analysis. Proteolytic peptide ions originating from unmodified protein sequences are identical in both samples, whereas different mass is observed for peptide ions containing the covalently modified amino acid residue. (**C**) Tandem MS or MS/MS. Following bottom-up MS analysis, proteolytic peptide ions (MS1) are exposed to fragmentation conditions that break the amide bonds, producing one out of two possible fragment ions for each broken peptide bond. The resulting fragment ions are annotated with increasing numbers from the N-terminus (b-fragment ions) or C-terminus (y-fragment ions). A mass difference is observed for fragment ions (MS2) containing the modified amino acid, thereby aiding identification of the modified amino acid residue.

Initial confirmation of a covalent binding mode is predominantly achieved through mass spectrometric (MS) analysis of the covalent protein–ligand adduct [37]. Here, validation of the covalent binding mode is based on the mass increase upon modification of an unbound protein with a covalent ligand, compared to the mass of the unbound protein (Figure 2). MS analysis provides confirmation of the biophysical binding event between the protein and the ligand but does not elucidate the bond layout. MS analysis is generally favored because it consumes a relatively low amount of material and is compatible with most protein targets. This versatile technique is not only used to validate a covalent binding mode but also in the discovery of new covalent ligands [40,43]. Detailed guidelines for mass spectrometric characterization (and quantification) of covalent protein–drug (metabolite) adducts are available elsewhere [37,75,76]. Generally, the covalent adduct is formed by incubation of protein with excess of inhibitor in an MS-compatible buffer, followed by a purification step such as liquid chromatography (LC) or gel electrophoresis [76,77]. MS analysis must be performed for the covalent adduct as well as the unbound protein, to confirm that the detected mass increase corresponds with covalent ligand modification. For top-down MS analysis (Section 2.1), the adduct is separated from the unbound protein/inhibitor under denaturing conditions to ensure all noncovalent interactions are disrupted prior to MS analysis of the intact protein–inhibitor adduct (Figure 2A). Alternatively, the adduct is submitted to proteolytic digestion with bottom-up MS analysis (Section 2.2) of the protein–derived proteolytic peptides to identify the peptide sequence modified by an irreversible covalent ligand (Figure 2B). Subsequent peptide ion fragmentation for MS/MS analysis (Section 2.3) can enable the identification of the modified amino acid residue (Figure 2C).

### 2.1. Top-Down MS

It should not be surprising that intact protein analysis by top-down MS is the most popular technique to validate covalent adduct formation with a wide variety of targets [63]: most (academic) drug discovery labs are equipped with an LC-MS system (Figure 2A), and sample preparation is relatively straightforward when the protein–ligand adduct is formed using recombinant purified protein. Benchmark protocols are composed by Donnelly and co-workers [77] for intact protein analysis by top-down MS. Generally, the unbound protein and protein (adduct) are ionized after denaturation and removal of unbound ligand on the LC, generating (positively or negatively) charged ions (z > 1) detectable by MS. The total mass of the parent protein or adduct is calculated by deconvolution of the charge states in the ionization envelope. It is important to note that sample preparation is conducted under denaturing conditions that ensure noncovalent interactions are disrupted. However, detection of noncovalently bound protein–ligand complexes is theoretically possible with native MS, with dedicated conditions to ensure noncovalent interactions are maintained [78,79]. The main practical limitations to intact protein analysis by top-down MS are the incompatibility with larger proteins (>50 kDa), proteins that ionize poorly [80,81], proteins that require MS-incompatible detergents or surfactants, and complex (cellular) mixtures that have not been enriched for the protein target: bottom-up MS analysis (Section 2.2) might be more suitable as ionization of peptides is often better.

Intact protein analysis by top-down MS is one of the less informative methods as it does not reveal the bond layout or identify which amino acid residue is covalently modified. Mitigation of covalent adduct formation by site-directed mutagenesis provides (indirect) evidence on which amino acid residue is modified, and is part of most covalent drug development workflows [63]. Intact protein analysis has recently been employed to validate covalent adduct formation with proteases [82,83,84,85], recombinant kinase domains [74,86,87,88], and other (potential) clinical targets [89,90,91]. Biophysical confirmation of covalent binding is also an important step in the ongoing industrial efforts to develop covalent kinase inhibitors with an improved selectivity/potency profile, as illustrated by intact protein analysis of covalent adducts between the BTK kinase domain and clinical candidates evobrutinib (Merck) [86], remibrutinib (Novartis) [87], and tirabrutinib (Ono Pharmaceutical/Gilead Sciences) [88].

*Covalent Fragment-Based Drug Development (FBDD).* Intact protein analysis has a prominent role in target-directed covalent fragment-based ligand discovery (FBLD). MS analysis is utilized to identify (cysteine-reactive) covalent ligands that serve as a starting point for medicinal chemistry optimization after validation of inhibitory properties associated with biophysical binding [37,38,39]. Kathman and co-workers developed an MS-based assay to screen mixtures of fragments containing a vinyl methyl ester (VME) warhead for covalent adduct formation with cysteine protease papain [43,85]. This assay has since successfully been employed for screening with cysteine-reactive covalent fragment libraries with mixed electrophile chemotypes [40,92], covalent ligand identification for E3 ligases [89,91], and several other recombinant protein targets [38,93].

**Figure 3:**
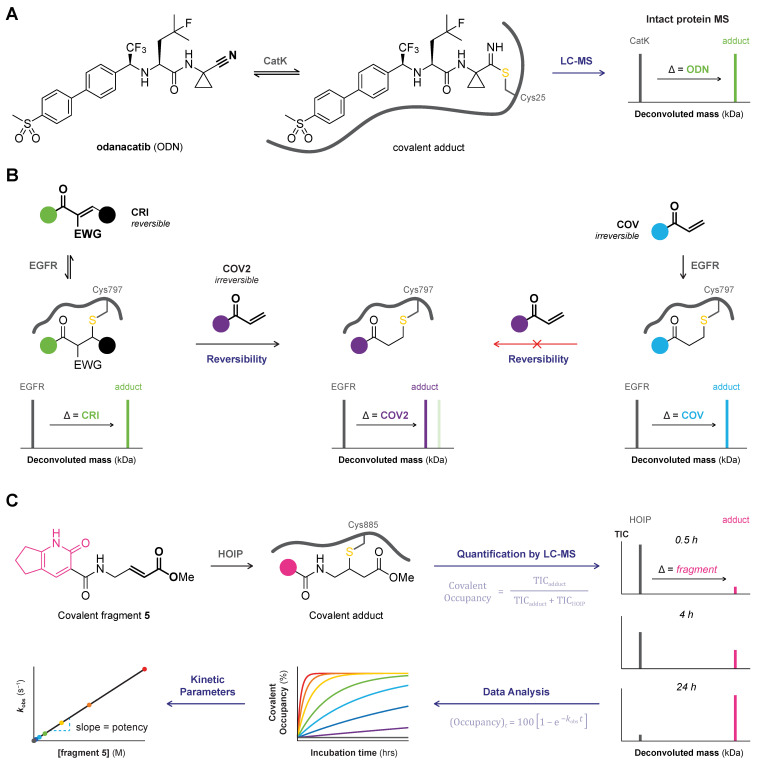
Biophysical covalent adduct detection with intact protein analysis by top-down MS. (**A**) Covalent CatK–ODN adduct formation between recombinant purified cathepsin K (CatK) and reversible covalent CatK inhibitor odanacatib (ODN) is confirmed by intact protein analysis [82]: the higher deconvoluted mass for the adduct compared to unbound CatK is in agreement with covalent ODN binding. (**B**) MS-based reversibility assay illustrated with recombinant purified EGFR^T790M/L858R^ mutant and reversible cyanoacrylamide-based inhibitor CRI [74]. Detection of covalent EGFR–COV2 adduct, rather than reversible covalent EGFR–CRI adduct upon competition with irreversible covalent chaser COV2, is indicative of a reversible binding mode. (**C**) Quantitative biochemical covalent occupancy assay illustrated for incubation of recombinant purified HOIP(RBR)^WT^ protein with excess covalent fragment **5** [89]. Adduct formation is detected by top-down MS and covalent occupancy is quantified from the total ion count (TIC) of the covalent adduct and unbound HOIP(RBR). The biochemical rate of covalent target engagement *k*_obs_ is calculated for each fragment concentration from the time-dependent covalent occupancy, which can be used to calculate the kinetic rate constant reflecting the binding efficiency of an irreversible covalent fragment (more details in [60,61]).

*Reversible binding mode.* The reversible covalent adduct of odanacatib bound to recombinant cysteine protease CatK [82] can be detected by top-down MS analysis (Figure 3A), provided that inhibitor concentration exceeds its steady-state equilibrium constant K_i_^*^ to ensure sufficient covalent occupancy [60]. The reversible covalent cTnC–levosimendan adduct was detected in endogenous thin and thick filament proteins extracted from porcine cardiomyofibrils [94]. However, sample preparation and denaturing conditions can induce inhibitor dissociation, and detection of the reversible covalent protein–inhibitor adduct is not possible if the covalent dissociation rate is relatively fast. Incubation of RSK2 with cyanoacrylamide CN-NHiPr failed to produce a detectable adduct [54,72] but this is highly context-dependent as covalent adducts with other cyanoacrylamides have since successfully been detected by top-down MS [57,74].

*Reversibility assays.* The Rauh group developed a top-down MS-based reversibility assay [74], illustrated for reversible covalent EGFR inhibitors bearing a cyanoacrylamide warhead (Figure 3B). EGFR kinase domain and reversible covalent inhibitor (CRI) are incubated to form the covalent EGFR–CRI adduct, followed by incubation with excess chaser COV2 (an irreversible covalent ligand selectively targeting the same amino acid residue, e.g., osimertinib or ibrutinib) that displaces the reversible covalent inhibitor, forming a covalent EGFR–COV2 adduct. Top-down MS analysis reveals a deconvoluted mass corresponding to the EGFR–COV2 adduct. Displacement is indicative of a reversible binding mode, as it is not possible to displace an irreversibly bound covalent inhibitor. Prerequisites to this reversibility assay are the availability of a selective irreversible chaser that targets the same amino acid residue and forms a covalent protein–chaser adduct that has a different mass than the protein–inhibitor adduct. This method has since been employed in the preclinical development of irreversible covalent BTK inhibitor tirabrutinib (ONO-4059) [88,95], which was resistant to chasing with ibrutinib. Indirect methods with MS detection of released free ligand upon induction of ligand dissociation (e.g., dilution, dialysis, washout, denaturation, competition) will not be further discussed here [54].

*Quantification covalent occupancy.* The research groups of House (Crick–GSK Biomedical LinkLabs) and Rittinger (Francis Crick Institute) recently reported a quantitative covalent occupancy assay for kinetic analysis of irreversible ligand binding to the RBR domain of HOIP, an RBR E3 ubiquitin ligase for which quantitative HTS activity assays are not available (Figure 3C) [89]. Time-dependent covalent occupancy was calculated from the total ion count (TIC) of the deconvoluted mass of covalent HOIP–fragment adduct relative to the unbound HOIP. LC-MS approaches were also employed to assess the potency of covalent KRAS^G12C^ inhibitors [37,96,97]. Differences in ionization efficiency of the unbound protein and adduct are only a minor concern as the protein size is significantly larger than the covalent ligand. Alternatively, indirect methods based on quantification of remaining unbound inhibitor (excess protein) [96] or unbound protein (excess inhibitor) [90,98] are employed to assess the biochemical rate of covalent target modification.

### 2.2. Bottom-Up MS

**Figure 4:**
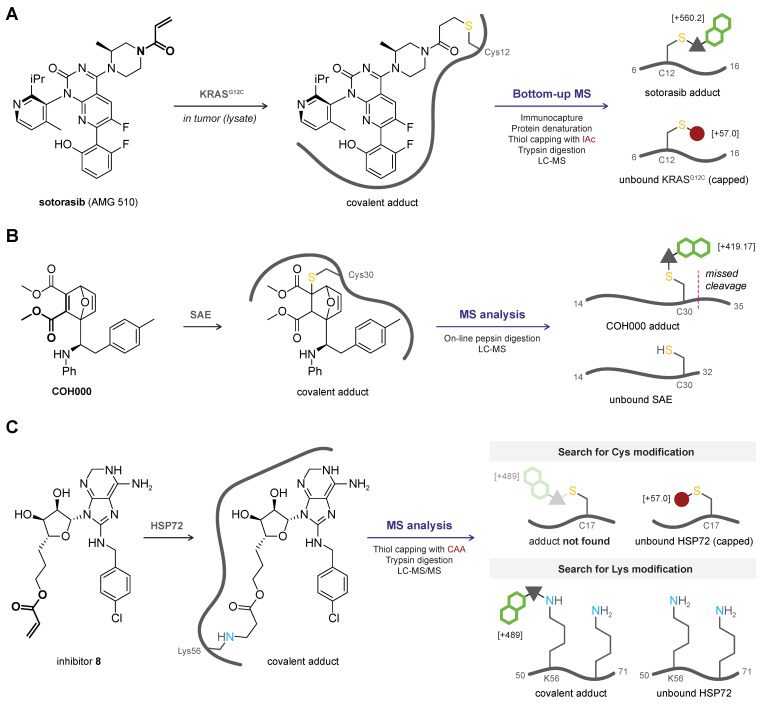
Bottom-up MS analysis of covalent protein–drug adducts. (**A**) Detection of covalent adduct formation for clinically approved covalent inhibitor sotorasib (AMG 510) with KRAS^G12C^ isolated from lysates originating from (in vitro or in vivo) treated tumor cells [18,99]. (**B**) Identification of covalent adduct formation between recombinant SUMO E1 (SAE) and covalent inhibitor COH000 [70] reveals unexpected modification of allosteric Cys30 rather than catalytic Cys173. Covalent modification of Cys30 interferes with pepsin-mediated digestion, generating longer adduct peptides than in the untreated control (missed cleavage). (**C**) Bottom-up MS analysis of recombinant HSP72 incubated with covalent acrylamide ligand **8** revealed covalent modification of Lys56 rather than catalytic Cys17 [71]. Data analysis focused on lysine modification resulted in detection of the simple adduct of L50–K71 peptide.

Bottom-up MS analysis (Figure 2B) is the preferred method to verify covalent adduct formation with large proteins (>50 kDa), with proteins that are poorly ionized, and for detection of covalent adducts in complex mixtures (e.g., cell lysates and samples from living organisms). A comprehensive overview of bottom-up MS methodologies is available elsewhere [76]. Generally, the protein–ligand adduct and the unbound protein are subjected to a thiol-alkylating reagent such as iodoacetamide (IAc) to cap free cysteine thiols (and sometimes lysine amines) with a carbamidomethyl group (+57.021 u), followed by trypsin- or pepsin-mediated proteolytic digestion (other proteases are also possible) [76]. The proteolytic peptides are separated by LC, ionized, and the peptide ions are detected by MS (Figure 4). Each parent peptide will be charged once or multiple times (z ≥ 1) to generate ionized peptides, and a database is used to correlate found m/z values with the predicted mass of various amino acid stretches. In the protein–ligand adduct, a peptide with a covalently modified amino acid residue appears along with a decrease or even disappearance of the (capped) unmodified peptide with the same sequence. Consequently, not only the covalent adduct is validated, but the peptide sequence containing the modified amino acid is also identified [76]. Optionally, sample preparation may involve purification by gel electrophoresis prior to capping and proteolytic digestion, to remove unbound ligand and MS-incompatible buffer components (e.g., surfactants, detergents) and enrich the sample for the desired protein (adduct). Proteolytic digestion ensures that only stable covalent adducts are detected but these harsh conditions also have a drawback: incompatibility with sensitive/labile functional groups. LC-MS detection of unbound inhibitor after digestion-induced inhibitor dissociation is commonly used to assess binding reversibility [49,55] but does not involve direct detection of the covalent adduct. Bottom-up MS analysis is compatible with complex mixtures and native systems. For example, covalent adduct formation of clinically approved covalent KRAS^G12C^ inhibitor sotorasib (AMG 510) in (in vitro or in vivo) treated tumor cells was detected [18,99] (Figure 4A). RAS proteins were isolated from lysates by immunocapture on anti-RAS beads, eluted proteins were denatured (8M urea), free thiols were alkylated with IAc, and proteins were digested with trypsin prior to mass spectrometric analysis. Bottom-up MS analysis has been employed to validate covalent adduct formation with oncogenic KRAS^G12C^ [18,99,100], proteases [82,84], and various other (potential) clinical targets [70,89].

The detected mass of the modified proteolytic peptide ions can correspond to the simple adduct (peptide + full ligand) but can be smaller if the ligand contains bonds sensitive to proteolysis (e.g., amide bonds), as is frequently seen for binding of ubiquitin(-like modifiers) [83]. Furthermore, covalent modification can block the proteolytic cleavage site, resulting in a missed cleavage and larger peptide sequences in the ligand-treated sample compared to the untreated (free protein) sample. This is exemplified by bottom-up MS analysis of the covalent adduct of SUMO-activating enzyme (SUMO E1 or SAE) with inhibitor COH000 [70] (Figure 4B): pepsin-mediated proteolytic peptide A14–L32 was found in the untreated sample, but the covalent modification of Cys30 in the COH000-treated sample blocked access to the pepsin cleavage site after Leu32, resulting in the simple COH000 adduct of peptide A14–N35 (missed cleavage). More importantly, bottom-up MS analysis was instrumental in the initial identification of the unexpected allosteric Cys30 modification: COH000 was expected to modify the catalytic (nucleophilic) Cys173 residue but only the unmodified A131–C185 peptide was found. The importance of careful interpretation of MS data is further illustrated by the work of Pettinger and co-workers [71]. Their covalent acrylamide ligand was designed to covalently target Cys17 in stress-inducible ATPase molecular chaperone heat shock 70 kDa protein 1 (HSP72) but no evidence of Cys17 labeling was found after proteolytic digestion and subsequent MS analysis (Figure 4C). Instead, they found evidence suggesting allosteric Cys267 was modified, but site-directed mutagenesis revealed that this modification only contributed to a minor covalent adduct and is not responsible for the inhibition of protein function. Finally, expanding the search to the modification of other nucleophiles (lysine) revealed the modified L50–K71 peptide. A reliable MS2 spectrum confirming Lys56 as the modified amino acid could not be obtained (details on MS/MS analysis in Section 2.3), but the unanticipated covalent modification of Lys56 driving the inhibitory activity was confirmed with the HSP72^K56A^ mutant.

*Reversible binding mode.* Direct detection of the proteolytic peptide modified with a reversible covalent inhibitor (CRI) or ligand can be challenging because denaturation and proteolytic digestion are known to promote CRI dissociation [49,54,55], and treatment with alkylating reagent to cap free thiols can block CRI rebinding. As such, modified peptides are more likely to be detected when thiol capping precedes denaturation and proteolytic digestion. It is possible to detect the modified tryptic peptides with bottom-up MS-based methods if the dissociation rate of the reversible covalent modifier is slow enough, as exemplified by the detection of proteolytic UCHL1 peptides modified with reversible covalent cyanimide IMP-1710 [84].

*Quantification of covalent occupancy.* Covalent target engagement is often quantified indirectly from the depletion of the unmodified proteolytic peptide in the treated sample relative to the untreated sample [96,97,101] as unbiased quantification of the (modified) proteolytic peptide can be challenging: ionization efficiency differences can occur following modification with a covalent ligand. The (LC-)MS/MS methods to overcome this bias will be discussed in the next section [100].

### 2.3. MS/MS or Tandem MS

**Figure 5:**
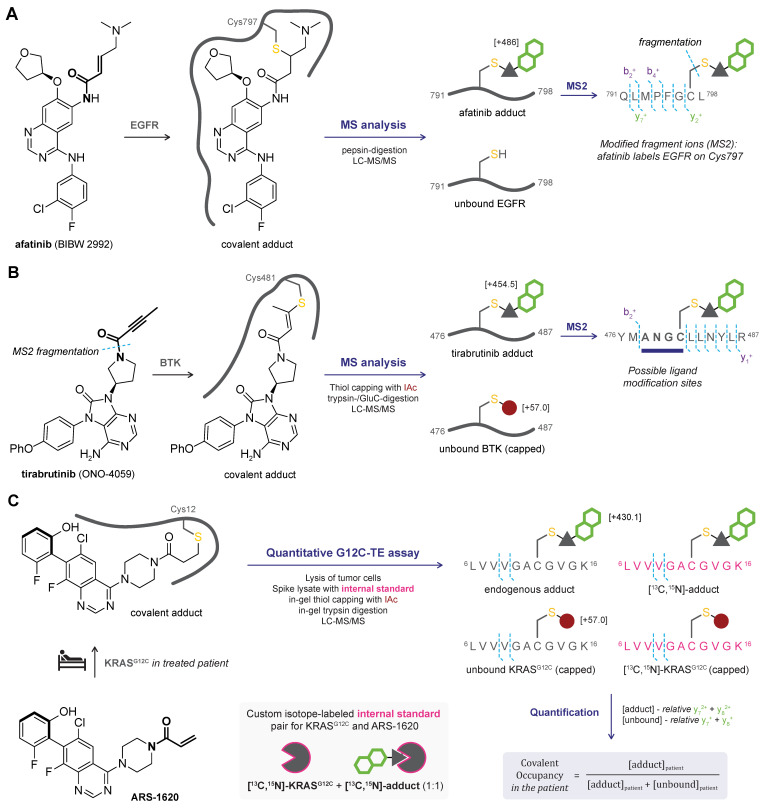
LC-MS/MS analysis of covalent protein–drug adducts. (**A**) Identification of the covalently targeted amino acid by afatinib (BIBW 2992) in purified kinase domain EGFR^T790M/L858R^ [14]. (**B**) Identification of covalently modified amino acids in recombinant BTK kinase domain by BTK inhibitor tirabrutinib (ONO-4059) [88]. MS2 detection only showed unmodified fragment ions and ligand fragmentation of unbound tirabrutinib. (**C**) Internally controlled quantitative KRAS^G12C^ Target Engagement (G12C-TE) assay illustrated with KRAS^G12C^ inhibitor ARS-1620 [100]. Lysates originating from clinical tumor biopsies are spiked with an internal standard: recombinant stable isotope-labeled KRAS^G12C^(1–169) internal standard consisting of a 1:1 mixture of free [^13^C,^15^N]-KRAS^G12C^ and covalent [^13^C,^15^N]-KRAS^G12C^–ARS-1620 adduct to calculate the in vivo covalent target occupancy from the relative abundance of fragment ions corresponding to endogenous or stable isotope-labeled adducts as well as unbound KRAS^G12C^.

Bottom-up MS analysis is frequently coupled to a subsequent MS analysis (tandem MS or MS/MS) to enable the identification of the covalently modified amino acid (Figure 2C). Specific precursor peptides are isolated after MS1 and subjected to collision/fragmentation conditions to generate charged fragment ions. The amino acid sequence of the precursor ion can be deduced from the mass of the fragment ions in MS2. For covalent adducts, fragment ions containing the covalently modified amino acid residue have a higher mass than fragment ions derived from the unmodified peptide, thus enabling the identification of the modified amino acid. Tandem MS procedures have frequently been used to identify the modified amino acid residue covalent inhibitors targeting different protein classes [18,22,70,71,82,84,89]. For the unambiguous assignment of the modified noncatalytic cysteine in the kinase domains affected by clinical irreversible covalent EGFR inhibitor afatinib (BIBW 2992) (Figure 5A) [14], LC-MS/MS analysis following pepsin-digestion proved very valuable. The modified Q791–L798 peptide ion was found in the MS1 spectrum, and MS2 data identified Cys797 as the modified amino acid. Moreover, the detection of a sulfurized afatinib fragment ion resulting from fragmentation of the C–S bond between the thiol and the cysteine β-carbon further confirmed covalent thiol addition to afatinib.

Covalent modification can (negatively) affect the ionization of peptide fragments, and it is not uncommon to only detect unmodified fragment ions (Figure 5B) [82,84,88]: LC-MS/MS analysis of the trypsin- and GluC-digested covalent adduct confirmed covalent binding of tirabrutinib (ONO-4059) [88] to the BTK Y476–R487 precursor peptide (MS1), but only unmodified fragment ions and fragmentation of the parent inhibitor were found in MS2. However, the unmodified fragment ions indicate that ligand modification occurred at one of the A478–C481 residues, of which Cys481 is the most nucleophilic residue. Interpretation of MS/MS data is usually tailored toward the modification of a specific amino acid class or even a single specific residue, searching only for modification of cysteine residues and performing MS2 for peptide ions containing the catalytic cysteine. Although it is generally safe to assume that covalent inhibitors bearing a thiol-reactive electrophile will target the nucleophilic catalytic cysteine residue, covalent modification of less reactive cysteines [70,91], or even unexpected amino acids, has been reported in exceptional cases [71].

*Reversible binding mode.* Limitations and challenges for reversible covalent ligands are similar to bottom-up MS analysis. Detection of the simple adduct and fragment ions containing the covalent ligand has been reported for reversible covalent ligands with a slow dissociation rate [84].

*Quantification of covalent occupancy.* Covalent in vivo target engagement of clinically approved KRAS^G12C^ inhibitor sotorasib (AMG 510) has been quantified from the percentage of modified KRAS^G12C^ peptide normalized to the total KRAS^G12C^ peptides in tumor cells recovered from treated mice [18]. However, caution is advised as this method does not take the possible effect of covalent modification on the ionization of the fragment ions into account. Quantitative covalent KRAS^G12C^ Target Engagement (G12C-TE) assays are typically indirect, using [^13^C,^15^N]-KRAS^G12C^ (peptide) as an internal control to determine the absolute level of unoccupied KRAS^G12C^ [90,98,101,102,103]. Cellular/biochemical occupancy is calculated from a comparison of unbound KRAS^G12C^ levels in the treated sample to the untreated control. However, these indirect methods are not compatible with the clinical development of solid tumor treatment because pretreatment or patient-matched reference biopsies are typically not available [104]. Scientists at Wellspring Biosciences [100] developed a direct, internally controlled quantitative MS/MS method for the accurate determination of target occupancy in FFPE (formalin fixed paraffin embedded) samples prepared from clinical tumor biopsies, without the requirement of pretreatment or untreated controls; illustrated for KRAS^G12C^ inhibitor ARS-1620 as a proof-of-concept study (Figure 5C). Here, tumor-derived lysates are spiked with an internal standard consisting of a 1:1 mixture of unbound [^13^C,^15^N]-KRAS^G12C^ and covalent [^13^C,^15^N]-KRAS^G12C^–inhibitor adduct, thereby enabling absolute quantification of endogenous unbound as well as modified KRAS^G12C^ peptide ions. The samples were then exposed to reducing conditions, with in-gel thiol capping with IAc and trypsin digestion of RAS proteins, before being submitted to LC-MS/MS analysis. Using the respective internal standard peptides, the ratio between endogenous unbound and ARS-1620-bound KRAS^G12C^ could be determined, allowing the calculation of in vivo covalent occupancy. This method is generally applicable for proteins with endogenous expression levels well above the limit of quantification by MS, but its application is practically limited by the production of recombinant stable isotope-labeled protein and the required generation of an isotope-labeled internal standard for each individual inhibitor.

## 3. Protein Crystallography

**Figure 6:**
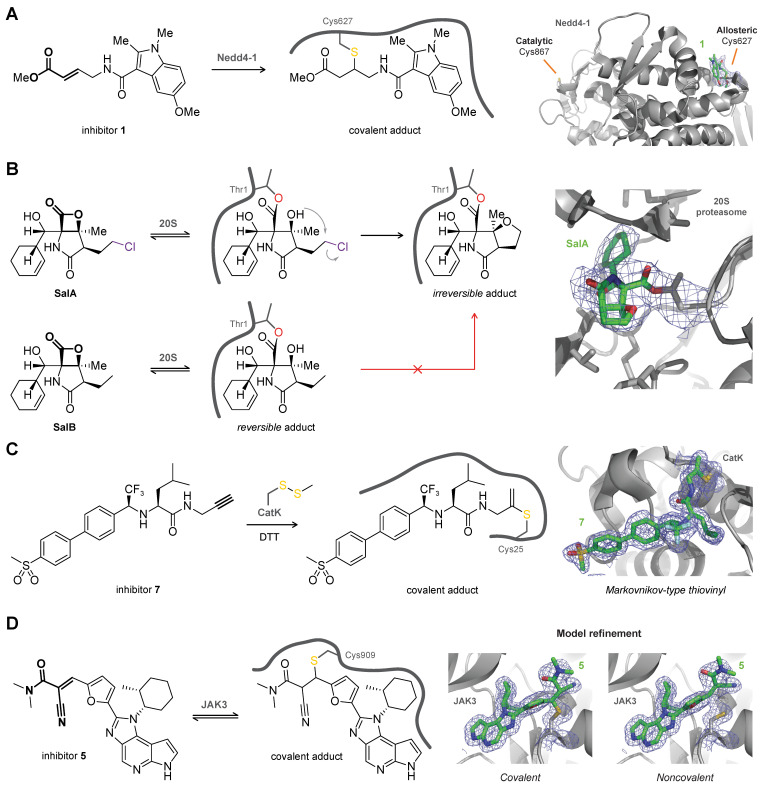
Structural binding information on covalent protein–drug adducts obtained by protein crystallography. (**A**) Identification of unexpected modified amino acid residue. Inhibitor **1** modifies ubiquitin E3 ligase Nedd4-1 on allosteric cysteine residue Cys627 rather than the more nucleophilic catalytic cysteine residue Cys867 (PDB: 5C91) [91]. (**B**) Protein crystallography of closely related 20S proteasome inhibitors salinosporamide A (SalA, NPI-0052, marizomib) and salinosporamide B (SalB) aids mechanistic understanding of their different binding modes [120]: SalA (PDB: 2FAK) [110] forms an irreversible adduct by ring closure with a chloride leaving group following the initial formation of the reversible covalent acyl ester with Thr1-OH. (**C**) Structural analysis confirms thiol–alkyne addition of catalytic Cys25 in human cathepsin K (hCatK) to the internal alkyne carbon on odanacatib derivative **7** (PDB: 6QBS) [82], forming a covalent adduct with a Markovnikov thiovinyl bond layout similar to the thioimidate adduct of hCatK with the odanacatib nitrile (PDB: 5TDI) [121]. (**D**) Refined electron density maps assuming covalent ligand binding (left) or noncovalent ligand binding (right) indicate a mixture of both states upon co-crystallization of reversible covalent cyanoacrylamide **5** with Janus kinase JAK3 (PDB: 5LWN) [122].

X-ray crystallography is a technique used to elucidate the 3D structure of crystalline compounds, from small molecules to (large) proteins [105]. Protein crystallography is the most informative technique discussed in this work: providing biophysical evidence on the covalent adduct along with detailed structural information on the modified amino acid residue and the bond layout of the protein–bound ligand. Covalent bonds between individual atoms are not directly observed: the distance between the individual atoms is detected, along with a continuous electron density, from which the likeliness that these atoms are involved in a covalent bond is determined, with the performance of orthogonal experiments (e.g., MS, mutagenesis) to validate covalency (Figure 6). One of the major practical drawbacks is the consumption of large amounts of highly pure soluble protein, and not all soluble proteins (or protein complexes) form suitably diffracting crystals (if any at all) [105]. In comparison: smaller protein amounts (of lower purity) are sufficient for less informative methods (e.g., MS). For protein crystals with appropriate diffraction, resolved macromolecular (ligand-bound) structures are deposited to the publicly accessible Protein Data Bank (PDB) [106,107], enabling other researchers to access this wealth of structural information. High-resolution structures of covalent adducts are available for various clinically approved TCIs including BTK inhibitors ibrutinib (PCI-32765, PDB: 5P9J) [108] and zanubrutinib (BGB-3111, PDB: 6J6M) [12], EGFR inhibitor afatinib (BIBW 2992, PDB: 4G5J) [14], and proteasome inhibitor bortezomib (PS-341, PDB: 2F16) [51]. The structural binding information can be used to gain insight into ligand binding driving target selectivity and/or reactivity [109,110,111,112] and can be combined with (covalent) docking studies [44,113,114,115] to aid the structure-based design of covalent ligands with improved potency and/or selectivity [22,45,116,117,118,119]. Structure-guided drug design approaches are employed to optimize the proximity of the electrophilic warhead to the nucleophilic amino acid [22]. The potency of clinical candidate ARS-1620 (PDB: 5V9U) was improved by surface groove occupation, resulting in enhanced interactions with the KRAS^G12C^ protein eventually leading to the development of clinically approved KRAS^G12C^ inhibitor sotorasib (AMG 510, PDB: 6OIM) [99].

A typical workflow starts with expression and purification of recombinant protein (domain), treatment with ligand, and screening hundreds of crystallization conditions to produce sufficiently large, singular crystals [105,123]. Suitable crystals of, hopefully, the covalent protein–ligand adduct are then fished, flash frozen, and exposed to an X-ray beam at a synchrotron. The atoms in a crystalline structure (at low temperature) are ideally stationary and the diffraction pattern is collected over different angles. The resulting intensities are then indexed to determine the space group and integrated into a dataset from which a 3D model can be determined [124]. As the dataset is a reciprocal space representation of the structure, the intensities need phases to actually solve the structure. Using one of the various phasing methods [125], an initial structure model and electron density can be determined. After various iterative rounds of model building and structure refinement, the electron density for the (covalent) ligand can, hopefully, be seen. The most reliable structural data is obtained when the model is first refined for the protein (based on the apo structure of the protein) and the remaining electron density is used to fit the ligand as this minimizes the bias for the inhibitor binding site [126].

For some proteins, apo structures of active, uninhibited enzymes may not be available for autoproteolytic/cannibalistic reasons (e.g., cysteine cathepsins) due to self-proteolysis or autodigestion [127,128]. Occasionally, one can obtain mixed crystals, consisting of free protein noncovalent protein–ligand complex and covalent protein–ligand adduct. This can decrease the quality and may impair detection altogether, thus requiring purification of the covalent adduct prior to crystallization. Alternatively, crystals of the free (apo) protein are allowed to form, before soaking in the (covalent) ligand, though this can result in mixed crystals [14] or cracking of the crystal. Soaking is popular in structure-based ligand screens as it conveniently sidesteps the optimization of crystallization conditions for each individual ligand [129]. However, soaking is not recommended for covalent drugs as the rigid crystalline protein can hinder the formation of a covalent adduct, especially if the crystalline apo-protein is in the incorrect conformation for ligand binding; if noncovalent ligand binding induces a conformational change before covalent adduct formation [130]; or if the crystalline protein has lost its catalytic activity essential for covalent adduct formation with mechanism-based inhibitors [48].

Non-crystallographers are advised to consult the works of Wlodawer and co-workers on the interpretation and critical evaluation of structural data [123,131]. The value for resolution is expressed as the smallest resolved distance (in Å = 10^−10^ m) in the structure model. Although the resolution applies to the whole map, parts of the structure may suffer from disorder and have high-temperature factors as a result [131,132]. It is important to realize that covalent adduct formation in these areas may not be reliably detected. Hydrogen atoms are not shown in most structures obtained by X-ray diffraction (XRD) as hydrogen atoms only weakly scatter X-ray beams: they only have one electron, which is always involved in a bond with another atom and are, therefore, not precisely localized at the usual resolution [133]. A low numeric value correlates with a high resolution: individual atoms (including some hydrogens) can be observed at <1.2 Å and most backbones and sidechains are clear at 2.5 Å, while only the general backbone can be solved at a resolution of 5 Å [124]. The average distance between a thiol atom and carbon atom in a covalent single C–S bond is 1.82 Å and cannot reliably be observed when the resolution at the ligand binding site is too low.

Protein crystallography has revealed unexpected modification of noncatalytic (allosteric) cysteine residues rather than the catalytic cysteine residue. Solving the crystal structure provided molecular insight into why a covalent E3 ubiquitin ligase Nedd4-1 inhibitor (PDB: 5C91) inhibits elongation of polyubiquitin chains but does not completely inhibit all catalytic activity [91]: the inhibitor targets allosteric Cys627 positioned at the substrate binding site rather than the more nucleophilic catalytic cysteine residue Cys867 (Figure 6A). Covalent modification of allosteric Cys30 in SUMO-activating enzyme (SUMO E1 or SAE) by COH000 rather than catalytic Cys173 (see Figure 4B) was validated by solving the X-ray crystal structure (PDB: 6CWY) [134]. Of note, protein crystallography reveals the protein–ligand complex or adduct that forms the best crystals but this does not have to be the most prevalent binding mode in solution: modification of a specific (unexpected) amino acid should be validated with orthogonal techniques to ensure that the modification is representative for ligand binding in solution. As such, protein crystallography is not a suitable technique for the quantification of covalent adduct formation.

Natural product salinosporamide A (SalA, NPI-0052, marizomib) is a clinically approved covalent 20S proteasome inhibitor with an irreversible binding mode, whereas closely related natural product salinosporamide B (SalB, NPI-0047) has a reversible binding mode (Figure 6B) [120]. Crystal structure analysis reveals that threonine Thr1 addition to the chloroalkyl β-lactone in SalA resulting in β-lactone ring opening is followed by intramolecular nucleophilic substitution to irreversibly form a stable cyclic tetrahydrofuran (THF) ring (PDB: 2FAK) [110] that cannot be formed with the β-lactone of SalB, thus aiding molecular understanding of the irreversible binding mode of SalA. Similarly, protein crystallography provided mechanistic insight on the superior selectivity of clinical multiple myeloma drug carfilzomib (PR-171, PDB: 4R67) [135] for the 20S proteasome over non-proteasomal proteases and why such selectivity is not observed for bortezomib (PS-341, PDB: 2F16) [51]. Carfilzomib forms a dual covalent adduct with the 20S proteasome, and the additional engagement of the Thr1 primary amine is specific for proteasomal proteins.

Protein crystallography provides valuable structural information on the bond layout of the covalently bound ligand. In our group, solving the crystal structure of ABP (activity-based probe) UbPrg with cysteine protease vOTU revealed an unexpected Markovnikov-type thiovinyl bond between the active site cysteine thiol in the protease and internal carbon of the alkyne in UbPrg (PDB: 3ZNH) [136]. This thiovinyl bond layout has since been observed for propargyl-containing ABPs targeting various cysteine proteases (listed in Table S1 of citation [83]) and for a small molecule CatK inhibitor (PDB: 6QBS) (Figure 6C) [82]. Active, mature CatK had to be treated with *S*-methyl methanethiosulfonate (MMTS) to prevent autodigestion in absence of competing substrate, which is more prone to occur at the high concentrations (>10 mg/mL) used for crystallography [127,128,137]. The thiomethyl protecting group is removed with a reducing agent (e.g., BME, TCEP, or DTT) to allow covalent adduct formation with the simultaneously added inhibitor [138].

*Reversible binding mode.* Crystallography does not involve stringent washing or (harsh) denaturing conditions that would promote ligand dissociation, thus being particularly suitable for the evaluation of reversible covalent ligands. Co-crystallization of clinical Hepatitis C virus (HCV) drug telaprevir (VX-950) with serine protease NS3/4A confirms the bond layout of catalytic Ser139 bound to the C-α carbon of the ketoamide warhead (PDB: 3SV6) [53]. Ligand interactions with frequently mutated protein sites provide a molecular basis for clinically occurring drug resistance. Importantly, covalent and noncovalently bound ligands may coexist in the crystal structure, as was demonstrated for reversible covalent cyanoacrylamide inhibitors targeting non-conserved cysteine Cys909 in an induced fit binding pocket of Janus kinase JAK3 (Figure 6D) (PDB: 5LWN) [122]. Other notable examples of reversible inhibitors are crystal structures of the first clinically approved proteasome inhibitor bortezomib (PS-341) [139] covalently bound to Thr1 of yeast 20S proteasome through the boronic acid moiety (PDB: 2F16) [51], COVID-19 drug nirmatrelvir (PF-07321332) forming a reversible covalent thioimidate adduct with catalytic cysteine Cys145 of SARS-CoV-2 M^pro^ protease in crystals formed by co-crystallization (PDB: 7RFW) or by soaking the apo crystal (PDB: 7RFS) [47], and the structure-based design of reversible covalent BTK inhibitors with tunable residence times [55].

## 4. Intrinsic Fluorescence/Absorbance

Covalent thiol addition can change the intrinsic spectroscopic properties of certain ligands and can be used to monitor covalent adduct formation in plate-based fluorescence/absorbance assays. Generating a chemical tool or an activity-based probe (ABP) by introducing a fluorophore or fluorescent leaving group to the ligand core will be discussed in more detail in Section 6. In this work, we will focus on ligands that do not require late-stage structural modifications because they contain a structural motif that has intrinsic spectroscopic properties [140], with a detectable change upon covalent adduct formation. This method is not generally applicable as there are strict structural limitations to the ligand core and nature of the electrophilic warhead. The main advantage of intrinsic spectroscopic methods is the compatibility with plate-based HTS assays, and catalytic activity is not required. The latter is directly a major drawback as it is impossible to discriminate between desired adduct formation with the intended cysteine thiol and undesired adduct formation with untargeted thiols present in the reaction buffer. This method always needs orthogonal validation as noncovalent binding events can also induce detectable changes in intrinsic fluorescence/absorbance [141].

**Figure 7:**
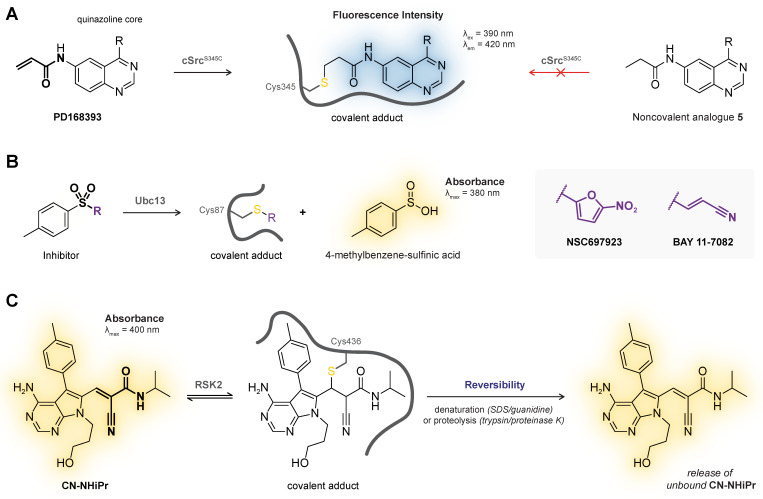
Direct detection of changes in intrinsic spectroscopic properties upon covalent thiol addition. (**A**) Thiol addition increases intrinsic fluorescence intensity of quinazoline and quinoline cores with an attached conjugated Michael acceptor [142]. Detection of increased fluorescence intensity upon adduct formation for irreversible covalent inhibitor PD168393 with cSrc^S345C^ but not for the noncovalent analogue. (**B**) Release of 4-methylbenzene-sulfinic acid results in a detectable absorption increase upon covalent adduct formation of inhibitors NSC697923 and BAY 11-7082 with Ubc13^WT^ [143]. (**C**) Intrinsic absorption in the UV-visible spectrum of *N*-isopropyl cyanoacrylamide CN-NHiPr decreases upon nucleophilic thiol addition [54]. The reappearance of signal upon protein denaturation or proteolysis-induced inhibitor dissociation is indicative of a reversible covalent binding mode.

The Rauh group [142] reported a plate-based assay for direct detection of covalent bond formation of quinazoline- and quinoline-based kinase inhibitors with an attached conjugated electron-deficient group such as an acrylamide warhead (Figure 7A). The unbound inhibitor exhibits weak fluorescence emission upon excitation due to photo-induced electron transfer (PET) from the quinazoline/quinoline core to the attached conjugated Michael acceptor. Covalent thiol adduct formation enhances the quantum yield and can thus be detected as an increase in the fluorescence emission as was illustrated for recombinant cSrc^S345C^ mutant (Cys345 mutation on an isostructural position to Cys797 in EGFR) with irreversible covalent quinazoline PD168393 but not with a noncovalent analogue [142].

Analogous to fluorogenic substrates that release a fluorescent group upon proteolytic cleavage [144], certain irreversible covalent inhibitors release a (detectable) leaving group upon covalent thiol addition (Figure 7B). This concept has been utilized to monitor covalent adduct formation of covalent inhibitors NSC697923 and BAY 11-7082 with E2 ubiquitin conjugating enzyme Ubc13 [143]: elimination of 4-methylbenzene-sulfinic acid upon covalent thiol addition can be monitored by an increase in absorbance in the UV-visible spectrum directly related to covalent adduct formation.

*Reversible covalent binding and reversibility assays.* Detection of intrinsic fluorescence is compatible with reversible covalent inhibition, as demonstrated by the Taunton group for thiol addition to reversible covalent kinase inhibitors bearing a cyanoacrylamide warhead [54]. They report that unbound *N*-isopropyl cyanoacrylamide CN-NHiPr has a strong intrinsic absorption in the UV-visible spectrum that disappears upon treatment with excess recombinant RSK2 kinase domain, which is consistent with nucleophilic thiol addition of Cys436 to the cyanoacrylamide warhead (Figure 7C). Reversibility could then be assessed by exposing covalent RSK2–CN-NHiPr adduct to denaturing conditions or proteolytic digestion to induce target dissociation: the reappearance of the absorption peak (and LC-MS detection of recovered unbound cyanoacrylamide) is in agreement with a reversible covalent binding mode.

## 5. Nuclear Magnetic Resonance (NMR)

Ligand-observed NMR analysis was the predominant method to detect covalent bond formation between enzyme and covalent inhibitor prior to the rise in popularity of MS analysis or protein crystallography [39]. A change in the chemical environment resultant from (non)covalent interactions causes a detectable change in the resonance frequency (typically reported as a ‘chemical shift’) and the coupling of nuclei with a nonzero nuclear spin (e.g., ^1^H, ^13^C, ^15^N) in the magnetic field of the NMR spectrometer. An overview of NMR spectroscopy principles for protein–ligand interactions can be found elsewhere [145,146,147]. Nowadays, NMR studies are employed in structure-based drug discovery and NMR screening for covalent (fragment) ligands [148,149] and have been used for ligand binding site mapping and structural elucidation of various covalent ligands. In macromolecular structure determination, NMR and protein crystallography can be complementary techniques [150,151], and NMR-resolved macromolecular (ligand-bound) structures are also deposited to the publicly accessible Protein Data Bank (PDB). Solution structures of covalent adducts have been deposited for compounds bound to protein targets [152,153,154] but also to minor groove duplex DNA: for example, the covalent adduct of chemotherapy drug mitomycin C (UGN-101) with a DNA 9-mer (PDB: 199D) [155] and the alkylating agent duocarmycin A covalently bound to a DNA 7-mer (PDB: 107D) [156]. Contrary to protein crystallography, NMR techniques are compatible with the characterization of binding mode reversibility by performing dialysis experiments [154], and ligand binding can be quantified to determine kinetic parameters (e.g., dissociation constant K_D_) [146,147,157,158].

Macromolecular structure determination typically involves multiple different NMR experiments to interrogate the different facets of the covalent adduct, but 2D NMR techniques that detect the scalar (through multiple bonds) correlation of protein atoms to ligand atoms (e.g., pulse programs based on (HSQC-)TOCSY, HMBC, or 2D-INADEQUATE) ultimately provide the most conclusive NMR-based evidence of a covalent protein–drug adduct because these correlations are exclusive to covalent adducts. Unfortunately, protein signals often overlap with ligand signals, making it practically impossible to confidently discern the correlation between a ligand atom and a protein atom in a covalent adduct because correlations of atoms residing in the same ligand/protein (that do not require a covalent adduct) overlap [153]. In this section, we will feature the two main detection principles: protein-observed NMR and ligand-observed NMR.

*Protein-observed NMR.* Protein-observed NMR experiments compare the signals originating from the protein in an unbound state to the protein–ligand complex; ligand binding changes the chemical environment of amino acids in the proximity of the ligand, inducing chemical shift perturbations that can be used to map the ligand binding site onto the protein structure [147,159]. Given the vast number of atoms in a protein and the low natural abundance of the most suitable isotopes (e.g., ^13^C, ^15^N), protein-observed NMR spectroscopy typically involves the production and purification of a uniform isotope-labeled protein along with recording a reference spectrum of the unbound protein to enable the assignment of peaks to specific protein atoms [160,161,162]. Protein-observed NMR experiments can be used to gain structural insight into ligand binding in solution, which is particularly useful for targets that are not compatible with crystallization or conformations that do not crystallize: for example, solution protein-observed NMR spectra revealed that noncovalent kinase inhibitor imatinib binds to c-Abl in a previously unidentified open state [163]. Furthermore, NMR experiments were employed to identify the binding site of covalent inhibitors of the *S. aureus* Sortase A enzyme (*Sa*-SrtA), and used to solve the structure of the covalent adduct (PDB: 2MLM, 6R1V) [153,154]. However, protein-observed NMR techniques are typically restricted to relatively small proteins (<50 kDa), and most techniques used in macromolecular structure elucidation (e.g., [^15^N,^1^H]-HSQC) cannot directly discriminate between a covalent or a noncovalent ligand. It is advisable to employ additional ligand-observed experiments or orthogonal techniques (e.g., MS analysis) for covalent adduct validation: protein-observed NMR experiments technically only provide indirect evidence on covalency [160,164].

*Ligand-observed NMR.* In ligand-observed NMR experiments, a change in the chemical shift of the ligand signals in the protein-ligand complex is compared relative to the ligand signals in the unbound ligand. The most popular ligand-observed NMR techniques for fragment screening (e.g., saturation transfer difference spectroscopy and its variants) are based on the NOE principle (proximity in space) and thus cannot discriminate between binding of covalent or noncovalent ligands [160,165]. ^1^H NMR chemical shift perturbations can be employed to distinguish unbound ligand from a covalent protein–ligand adduct, and support the identification of the adduct isoform [166]. In addition to validation of covalent adduct formation, 1D ^1^H NMR approaches enable indirect quantification of covalent occupancy by integration of the disappearing unbound ligand signals [39,167]. However, ^1^H NMR experiments are typically only performed for adduct formation with small molecule thiol reagents (e.g., GSH) as overlapping background signals originating from protein hydrogens limit the practical application [39,167].

Direct detection of the covalent protein–ligand adduct by ligand-observed ^13^C NMR experiments is a more feasible approach but requires chemical synthesis of the ligand with a ^13^C-labeled warhead to improve the signal over the background; otherwise, the naturally occurring ^13^C signals in the ligand will be lost among those originating from the protein. The ^13^C chemical shift perturbations of adjacent carbons in the electrophilic warhead can be significant upon covalent thiol modification, especially compared to the less pronounced shifts induced by noncovalent binding interactions [168]. Detection of chemical shift perturbations of (isotope-labeled) epoxy succinyl peptides upon cysteine protease papain binding was successfully utilized to detect the covalent adduct along with identification of the covalent modification site [169]. Moreover, ^13^C NMR APT (attached proton test) experiments can be indicative of covalent adduct formation with unsaturated electrophiles (e.g., acrylamide): the phasing of the vinyl carbon adjacent to the reactive carbon in the unbound acrylamide is negative (CH) but is positive (CH_2_) in the covalent adduct.

Future application of ligand-observed NMR may be extended beyond the common ^13^C NMR and ^1^H NMR without the chemical introduction of an isotope-labeled atom for warheads bearing naturally abundant reactive atoms compatible with NMR (e.g., ^31^P or ^19^F in fluorophosphonates). ^11^B NMR has been employed to detect the tetrahedral adduct of boronic acid covalently bound to Ser195 in the serine protease trypsin [170]. This label-free approach has only been employed to study model reagents [171], but there still are seemingly unexplored opportunities for covalent adduct detection of boronic acid-bearing inhibitors (e.g., proteasome inhibitor bortezomib) with their pharmaceutical target.

**Figure 8:**
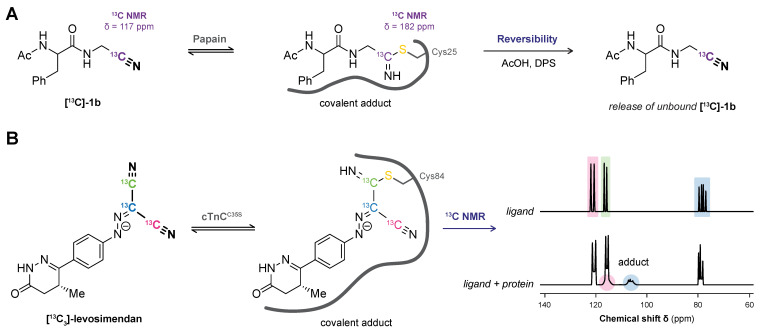
Ligand-observed ^13^C NMR detection of covalent protein–drug adducts. (**A**) Chemical shift perturbation of the electrophilic ^13^C-labeled carbon in unbound nitrile [^13^C]-**1b** relative to the thioimidate ester adduct provides evidence of a covalent papain–nitrile adduct [172]. Detection of unbound nitrile upon treatment with glacial acid (AcOH) and thiol-trapping reagent 2,2′-Dipyridyldisulfide (DPS) is indicative of a reversible covalent binding mode. (**B**) Ligand-observed NMR studies with ^13^C-labeled levosimendan provide evidence for reversible covalent binding to a cysteine thiol in cardiac troponin C (cTnC) [176].

*Reversible inhibition and reversibility assays.* An important advantage of NMR-based detection of covalent adducts is the compatibility with reversible covalent inhibitors. Especially when the covalent adduct is too short-lived to be isolated or detected due to rapid inhibitor dissociation under MS/sample preparation conditions as NMR enables detection in (aqueous) solution [54]. Ligand-observed ^13^C NMR analyses were already performed in 1986 [172] to obtain evidence for the formation of a thioimidate ester adduct between a nitrile ligand and the active site sulfhydryl of cysteine protease papain (Figure 8A). Incubation of active papain with the [^13^C]-labeled nitrile inhibitor resulted in the appearance of a resonance signal at 182 ppm in ^13^C NMR in accordance with a covalent thioimidate ester adduct. The rapid disappearance of the thioimidate signal and increase of unbound inhibitor signal (~117 ppm) was detected upon treatment of the covalent adduct with glacial acid (AcOH) and thiol-trapping reagent 2,2′-Dipyridyldisulfide (DPS), indicative of a reversible covalent binding mode. Similar ligand-observed NMR studies have been performed to provide evidence for reversible covalent adduct formation of cathepsin K with a [^13^C, ^15^N_4_]-diacylhydrazine [173] and papain with a [^13^C]-cyanimide [174]. A more recent example of ligand-observed NMR analysis aided elucidation of the binding mode of Ca^2+^ sensitizer levosimendan (Figure 8B), a clinical drug for heart failure treatment whose exact mechanism of action remained elusive for over 20 years after its discovery. Formation of a thioimidate bond between the electrophilic malonitrile moiety and cardiac troponin C (cTnC) was always assumed to have an important role [175], but evidence for this reversible covalent binding mode was finally provided in 2016 by employing [^13^C_3_]-levosimendan in ligand-observed ^13^C NMR studies [176]. The disappearance of unbound [^13^C_3_]-levosimendan signals (~120 ppm) along with the appearance of new signals (~160 ppm) is in agreement with predicted chemical shifts for a thioimidate adduct between the electrophilic malonitrile moiety on levosimendan and a cysteine thiol in cTnC. Lack of adduct in presence of cTnC^C84S^ but not cTnC^C35S^ validates Cys84 as the covalently modified amino acid residue [29].

## 6. Activity-Based Protein Profiling (ABPP)

**Figure 9:**
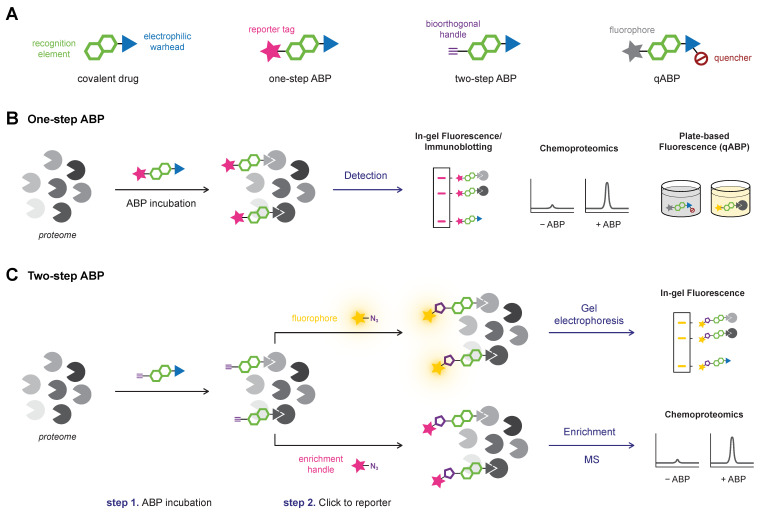
Strategies for covalent adduct detection with drug-derived activity-based probes (ABPs). (**A**) General design principle for covalent drug-derived ABPs. A fluorophore, detection tag, or enrichment handle is introduced onto the parent covalent drug bearing a recognition element and a covalent warhead. (**B**) Detection of covalently modified proteins with one-step ABPs. Proteome is treated with one-step ABP, proteins are resolved by gel electrophoresis or affinity purification, and modified targets are detected by in-gel fluorescence or immunoblotting or by chemoproteomic evaluation. (**C**) Detection of covalent adducts with two-step ABPs. Proteome is incubated with a two-step ABP bearing a small bioorthogonal handle (*step 1*), followed by bioorthogonal coupling of a fluorophore, detection tag, or enrichment handle (*step 2*), with subsequent analysis as shown for the one-step ABPs.

Activity-based protein profiling (ABPP) is a chemical biology technique that employs covalent activity-based probes (ABPs) to characterize covalent enzyme modification in relevant biological systems (e.g., live cells, in vivo) [104,177,178,179,180]. Pioneered in the labs of Cravatt [181] and Bogyo [182], ABP entailed a reactive group with a detection tag (e.g., fluorophore, radiolabeled isotope), or enrichment handle (e.g., biotin), that covalently modified catalytic serine/cysteine residues in active and uninhibited enzyme [178,183]. This general structure design is mostly maintained in modern ABPs that typically comprise an electrophilic moiety that forms a covalent bond with a nucleophilic amino acid residue, a reporter group (e.g., fluorophore, enrichment handle, bioorthogonal handle) to detect the covalent adduct, and optionally, a recognition element for target/class-selectivity (Figure 9A). 

Nowadays, ABP development is not limited to catalytic amino acid residues: a wide range of ABPs is available from general residue-specific agents (e.g., iodoacetamide (IAc)-based thiol-alkylating reagents for cysteines [184]) and class-specific ABPs (e.g., fluorophosphonate-based probes for serine hydrolyses [177], ubiquitin-based probes for DUBs [185], ATP-based probes for kinases [186]) to target-selective ABPs [36] (e.g., ibrutinib-based ABPs for BTK [11]). The field has since expanded beyond truly activity-based probes: ABPs targeting noncatalytic residues also label catalytic inactive mutants, thus not requiring catalytic activity [11]. ABPP has a prominent role in covalent drug discovery [178,187,188]: not only for identification of new covalent hits in covalent (fragment) screening [189] but also to identify cellular/in vivo covalently modified (off-target) proteins, thereby derisking covalent inhibitor development [2,190]. The latter is emphasized by the recent work of van Esbroeck et al. [191]: multiple off-target lipases targeted by fatty acid amide hydrolase (FAAH) inhibitor BIA 10-2474 were identified by competitive ABPP, providing a possible explanation for the clinical neurotoxicity with a lethal outcome for one of the human subjects in the phase I clinical trial (2016). This tragic example highlights why the identification of potential covalent off-target modifications by (competitive) ABPP is recommended to be an integral part of the early-stage covalent drug development [190].

Drug-derived ABPs are designed in two flavors: one-step ABPs (Figure 9B) and two-step ABPs (Figure 9C). One-step ABPs are generated by the introduction of a (fluorescent) detection tag or an enrichment handle onto the parent drug by chemical synthesis. The tag or handle is introduced in a position that does not interfere with target binding, as indicated by structural data (e.g., crystal structure, docking simulations) or SAR analysis. The introduction of a large tag/handle can modify ligand reactivity, target selectivity, as well as cell permeability [192]. Similar to one-step ABPs, two-step ABPs are generated from the parent drug but now a small and nonperturbing bioorthogonal handle is introduced, to which the actual detection group (fluorophore, enrichment handle) is clicked in the second step (Figure 9C). This bioorthogonal handle is less likely to have a pronounced effect on ligand selectivity, which is why evaluation of a two-step ABP is recommended in an early stage of covalent drug development to identify potential off-target effects [32,190]. Here, the proteome is treated with the two-step ABP bearing a small bioorthogonal handle (*step 1*), followed by the coupling of a relatively large detection tag or enrichment handle (*step 2*). Traditionally, the coupling reaction employs the Huisgen Copper-catalyzed Alkyne–Azide Cycloaddition (CuAAC) reaction between alkynes and azides (‘Click’ chemistry) [193,194], but alternative bioorthogonal reactions are available [192,195] such as the Strain-Promoted Alkyne–Azide Cycloaddition (SPAAC) between strained alkynes and azides [196], or the Inverse Electron Demand Diels Alder (IEDDA) reaction between (fluorogenic) tetrazines and strained dienophiles [197]. Two-step ABPs enable incubation in the native environment and are more likely the retain the membrane-penetrating properties of the parent inhibitor and are thus compatible with in situ and in vivo applications [104,198]. The success of this approach has recently been illustrated for inhibitors with various targets [32], among them the BTK inhibitor ibrutinib [11,190], JAK3 inhibitor ritlecitinib (PF-06651600) [199], KRAS^G12C^ inhibitor adagrasib (MRTX849) [103], and anti-obesity drug orlistat [200]. Two-step clickable ABPs facilitate the coupling of a dual biotin/TAMRA-azide, allowing both in-gel fluorescence scanning for the TAMRA fluorophore and immunoblotting for the biotin tag in gel-based evaluation, and the biotin tag can also be utilized as an enrichment handle in chemoproteomic evaluation. The success of this dual approach is illustrated by clickable two-step ABPs equipped with a bioorthogonal alkyne handle: ABP PF-06789402 based on the scaffold of JAK3/TEC family kinase inhibitor ritlecitinib (PF-06651600) [199], and ABP selinexor-yne derived from clinically approved covalent XPO1 inhibitor selinexor (KPT-330) [201].

Altogether, ABPP is a powerful tool to identify (un)desired covalent modification in a relevant biological setting. In this work, we will discuss the detection of the covalent adduct in whole proteome with gel electrophoresis platforms (Section 6.1), chemoproteomic platforms (Section 6.2), and homogeneous (plate-based) platforms (Section 6.3).

### 6.1. Gel Electrophoresis Platforms (In-gel Fluorescence, Immunoblotting)

**Figure 10:**
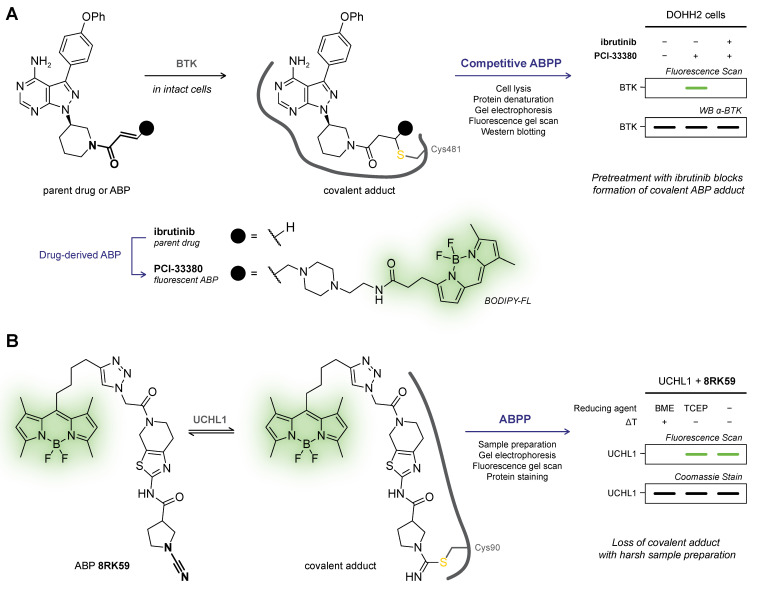
Gel electrophoresis platforms for covalent adduct detection with drug-derived ABPs. (**A**) Competitive ABPP. The fluorescence scan for the BODIPY-FL fluorophore reveals that BTK labeling in DOHH2 cells by cell-permeable one-step fluorescent ABP PCI-33380 is precluded by pretreatment with irreversible covalent parent inhibitor ibrutinib [11]. (**B**) The fluorescent signal originating from reversible covalent UCHL1–8RK59 adduct is not observed using harsh sample treatment prior to gel electrophoresis (boiling in presence of reducing agent BME) but can be observed using milder conditions (sample preparation in presence of reducing agent TCEP) [207]. Covalent adduct formation is validated using intact protein analysis by top-down MS.

Gel electrophoresis platforms were among the earliest ABPP methods to interrogate enzyme activity in complex mixtures and are still a common method for rapid evaluation of inhibitor specificity [178]. A typical workflow (Figure 9B) involves incubation of recombinant protein or a whole proteome (e.g., cell lysate) with a one-step ABP followed by sample preparation under denaturing conditions (e.g., heating in presence of a reducing agent such as BME or TCEP) to simultaneously remove unreacted ABP and promote dissociation of noncovalent complexes. Then, the treated proteome is submitted to gel electrophoresis, and covalent adducts are visualized by in-gel fluorescence scanning for the fluorophore (e.g., TAMRA, Cy5, BODIPY) [11,199,201] or immunoblotting for a reporter tag or enrichment handle (e.g., biotin, GST, His) [202], with a band appearing at the adduct mass (kDa). Gel-based ABP analysis is fast but is less informative than chemoproteomic approaches (discussed in Section 6.2). Identifying the exact protein target in a proteome can be challenging as proteins of similar mass may overlap on the gel, which may be addressed by comparative ABPP with knock-out cell lines [203,204]. Competitive ABPP experiments are typically conducted to validate that the ABP has the same specificity as the inhibitor [11,199,205,206]: treatment with parent BTK inhibitor ibrutinib (PCI-32765) precludes labeling with cell-permeable fluorescent ABP PCI-33380 (Figure 10A) [11]. The modified amino acid can be identified indirectly by treatment of (recombinant) protein with a single point-mutation [11,83,206], or by competitive labeling of the parent inhibitor with a validated residue-selective ABP (that is not derived from the inhibitor of interest) [178,207].

Most covalent drugs are too small to cause a detectable shift in electrophoresis upon covalent adduct formation, thus requiring modification with a detection group. Nevertheless, a DNA electrophoretic mobility shift assay [208] has been employed to validate the covalent binding mode of lurbinectedin (PM01183) to naked DNA, despite its relatively small mass (785 Da).

*Reversible inhibition.* ABPs bearing a reversible covalent warhead are compatible with gel-based analysis, as illustrated with cyanimide-based ABPs IMP-1710 [84] and 8RK59 [207] targeting deubiquitinating enzyme UCHL1 (Figure 10B). Importantly, reaction conditions required careful optimization as the fluorescent covalent adduct could not be detected after sample preparation under denaturing conditions: heating BODIPY-labeled UCHL1–8RK59 adduct to 94 °C in presence of reducing agent β-mercaptoethanol (BME) promoted covalent target disengagement [207]. Subsequent chemoproteomic evaluation with 8RK64, an alkyne-bearing derivative of 8RK59, revealed enrichment of not only UCHL1 but also protein deglycase PARK7/DJ1, an attractive target in Parkinson’s disease with a similar molecular mass that overlaps with UCHL1 by gel analysis. The discovery of this off-target modification has since aided the development of selective chemical tools to study PARK7 activity [209].

*Quantification of covalent occupancy.* Direct gel-based strategies are typically used for qualitative (visual) identification of binding partners in cellular proteomes as low throughput gel electrophoresis strategies are associated with large error margins originating from deviations in gel loading volumes and protein distribution on the gel. Our group reported a direct quantitative approach to calculate relevant kinetic parameters from time-dependent covalent occupancy of purified recombinant cysteine protease USP16 with irreversible covalent Rho-Ub-ABPs [83]. Cellular JAK3 occupancy after pretreatment with ritlecitinib (PF-06651600) was assessed with two-step ABP PF-06789402 [199]. Lysate was treated with biotin/TAMRA–azide, enriched for ABP-modified uninhibited proteins by pulldown with streptavidin beads, and resolved by SDS-PAGE gel electrophoresis to quantify the remaining uninhibited JAK3 by immunoblotting. A popular though indirect approach in the preclinical development of BTK inhibitors is to derive inhibitor target engagement from the fluorescent ABP labeling of the remaining unbound protein: inhibitor-treated proteome is incubated with a target-selective fluorescent ABP (not necessarily derived from the parent inhibitor) that only binds to unbound BTK, and ABP-bound BTK is quantified by in-gel fluorescence. This approach has been successfully applied with irreversible BODIPY-labeled BTK-selective ABP PRN-933 to assess occupancy of reversible covalent BTK inhibitor rilzabrutinib (PRN-1008) in human PBMCs [49], and in the development of reversible covalent BTK inhibitors with irreversible ABP PP-BODIPY [55]. Competition with general thiol-reactive ABPs (e.g., IAc–alkyne, TMR–maleimide) is of little use in gel-based ABP analysis as blocking a single cysteine residue will not perturb the ABP from binding to other available cysteine residues in the same protein, thus still resulting in a detectable signal on the gel. Importantly, blocking adduct formation with a selective irreversible ABP provides indirect evidence on the ligand binding site [74] but is not suitable to identify the modified amino acid by itself as this also could be a(n) (allo)steric effect.

*Scintillation autoradiography (fluorography).* Drug-derived ABPs bearing a radioisotope tag (e.g., ^125^I) used to be the primary mode for the detection of catalytically active cysteine proteases [210,211]. Nowadays, radiolabeled inhibitors prepared for in vivo ADME (Absorption, Distribution, Metabolism, and Excretion) studies and PK (pharmacokinetic) profiling [212] are occasionally employed as radioactive ABPs where the radioactive atom (typically ^14^C or ^3^H) serves as a small, non-perturbing tag. Radioactivity originating from the radiolabeled covalent adduct is detected after removal of unbound and noncovalently bound radiolabeled inhibitor by gel electrophoresis on polyacrylamide/SDS-PAGE gels (fluorography) [213] or by filtration with stringent washing (liquid scintillation counting) [213,214]. This technique has recently been employed to validate the covalent adduct formation of neratinib (HKI-272) with HER2, using neratinib-derived ABP [^14^C]HKI-272 ([^14^C]-25o) on recombinant HER2 cytoplasmic domain or in intact BT474 cells [16,215]. Similarly, in vivo covalent alkylation of hemoglobin by RRx-001 (ABDNAZ) in red blood cells from various species was detected using radiolabeled ABP [^14^C]RRx-001 [216]. Finally, scientists at Takeda Pharmaceutical developed a direct competitive ligand binding assay to quantify covalent occupancy and determine the inhibitory potency (*k*_inact_/K_I_) of irreversible EGFR inhibitor canertinib (CI-1033) [214]: time-dependent radioactivity originating from the covalent adduct with radiolabeled ABP [^3^H]CI-1033 (after filtration to remove unbound ABP) was quantified by liquid scintillation counting.

### 6.2. Chemoproteomic Platforms

Chemoproteomic protein profiling is a sensitive MS-based ABPP technology predominantly employed to evaluate the proteome-wide selectivity of covalent inhibitors and identify (undesired) cellular targets for covalent modification in complex mixtures (e.g., cell lysate, live cells, tissue) [38,104,190,191,217,218,219]. A general chemoproteomic procedure involves incubation of the proteome with ABP, coupling to an enrichment handle (e.g., biotin–azide, Figure 9C), and pull-down to enrich for ABP-modified proteins on beads. Stringent washing is performed to remove noncovalently bound proteins and eliminate nonspecific binders, followed by bottom-up MS/MS analysis of modified proteins. Quantification of relative protein abundance can be achieved with label-free quantitative methods comparing relative changes in two (or more) individual biological samples [198,219,220,221].

**Figure 11:**
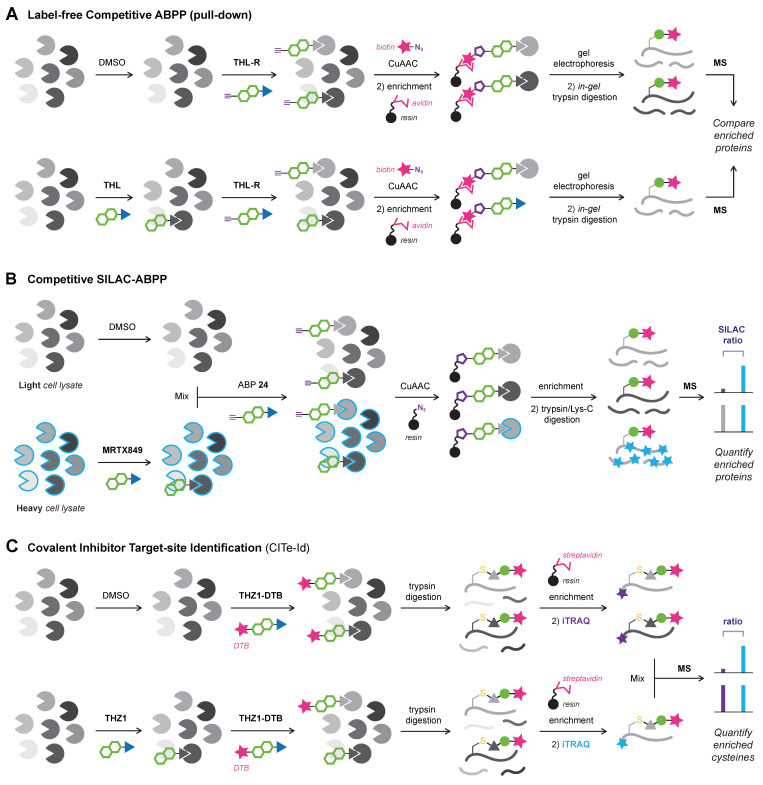
Chemoproteomic approaches to identify covalently modified (off-)target proteins in whole proteomes. Pretreatment with inhibitor blocks drug-derived ABP binding and protein enrichment, resulting in a lower abundance of target protein compared to the DMSO-treated (control) sample. (**A**) Label-free protein target detection in pull-down experiment with drug-derived ABP THL-R to identify in situ protein targets of orlistat (THL) in HepG2 cells [200]. ABP-bound proteins are enriched on avidin-agarose beads and submitted to bottom-up MS/MS evaluation. (**B**) Multiplexed detection of cellular protein targets of KRAS^G12C^ inhibitor adagrasib (MRTX849) in a competitive SILAC-ABPP experiment [103]. NCI-H358 cells cultured in normal (light) or isotope-labeled (heavy) medium (metabolic stable isotope-labeling) are incubated with DMSO or adagrasib, and the mixture of heavy and light lysate is then treated with drug-derived two-step ABP 24. ABP-bound proteins are enriched on azide-labeled agarose beads and submitted to bottom-up MS/MS for identification and relative quantitation of enriched protein abundance (SILAC ratio). (**C**) Identification and quantitation of novel protein targets for inhibitor THZ1 in a CITe-ID experiment [231]. Cell lysates preincubated with DMSO (control) or THZ1 (inhibitor) are treated with drug-derived desthiobiotinylated ABP THZ1-DTB and enriched for DTB-modified proteolytic peptides. Primary amines are labeled with a unique isobaric iTRAQ reagent in each sample, and samples are combined for multiplexed RP-SAX-RP MS/MS analysis.

The majority of chemoproteomic formats are indirect, detecting proteins modified by a general thiol-alkylating or cysteine ABP (e.g., IAc–alkyne) [189,222,223] in presence and absence of the covalent inhibitor of interest, from which inhibitor-binding is deduced [178,187,223]. For this purpose, classic broad-spectrum reactivity ABPs targeting various amino acid residues and protein classes are (commercially) available [36,38,186,223]. In particular, the isoTOP-ABPP (isotopic Tandem Orthogonal Activity-based Protein Profiling) platform is an established indirect competitive method to simultaneously identify (off-)target modified proteins in whole proteomes together with the exact site of protein modification [2,190,222,224]. The role of predominantly indirect competition methods in (fragment-based) covalent drug development has been reviewed before [36,38,225]. In this work, we will focus on the (less prevalent) direct approaches in which the ABP is derived from the parent inhibitor. Direct (competitive) chemoproteomic approaches with drug-derived ABPs have the potential to identify lower abundance protein targets and can overcome the bias in global cysteine reactivity experiments with general thiol-reactive ABPs: inhibitor binding is only detected if the amino acid residue is targeted by the competing ABP, even though the inhibitor might be interacting with other amino acid residues [103,198]. Such targetable, or ‘druggable’, cysteines in human proteins were recently collated in the publicly available curated repository CysDB [226]. The success of a direct approach is illustrated by the FDA-approved anti-obesity drug tetrahydrolipstatin (THL, orlistat) (Figure 11A): MS analysis following pull-down of modified proteins in cancer cell lines treated with two-step ABP THL-R did not only confirm binding to fatty acid synthase (FAS) but also identified other (off-)target proteins that aid its early development as an anticancer agent [200].

High-throughput proteomic methods may involve metabolic or chemical labeling with stable heavy isotopes prior to MS analysis to enable absolute protein quantification and multiplexed measurements (mix of multiple samples/reaction conditions) minimizing run-to-run deviations [221,227]. The popular SILAC-ABPP platform (Figure 11B) is a metabolic isotope-labeling methodology, thus being restricted to stable cell lines as generating isotope-labeled controls is challenging for tissue or primary cell line samples [228]. SILAC-ABPP combines ABPP with SILAC (stable-isotope labeling by amino acids in cell culture) [229,230] to assess the identity of covalent modified proteins. Cells are cultured in normal (light) or isotope-labeled (heavy) medium, treated with DMSO or ABP, and mixed after lysis. Modified proteins are detected by bottom-up LC/LC-MS/MS analysis after enrichment for covalent protein–ABP adducts. The isotope labeling of the proteome is crucial to calculate the SILAC ratio compared to the untreated sample (proteins with SILAC ratios ≥ 3–5 are designated as targeted). SILAC-ABPP analysis with an ibrutinib-derived ABP identified established off-target kinases as well as specific non-kinase targets from structurally and functionally diverse protein families in Ramos cells, including the uncharacterized protein FAM213A [190]. Typically, complementary competitive SILAC-ABPP experiments are performed to ensure that the drug-derived ABP has the same selectivity as the parent inhibitor: over 400 proteins were identified in a SILAC experiment with an adagrasib-derived ABP (Figure 11B) but only KRAS^G12^ significantly decreased upon pretreatment with clinically approved KRAS^G12C^ inhibitor adagrasib (MRTX849) [103].

Chemical isotope-labeling methods are compatible with samples that are not amendable for SILAC, such as endogenous (human) tissue samples [104], as chemical isotope-labeling can be performed during sample preparation [221,232]. These methods are typically indirect, employing classic broad-spectrum reactivity ABPs. Among the most popular methodologies are isoTOP-ABPP [222,224,233] using isotope-labeled TEV protease-cleavable Click reagents (TEV tags), isoDTB-ABPP employing isotopically-labeled desthiobiotin (isoDTB) tags [234], rdTOP-ABPP [235] employing stable-isotope diMe labeling of primary amines (peptide N-terminus and lysine ε-amino group) [227,236,237,238,239], and TMT-ABPP [240] employing tandem mass tags (TMT) such as isobaric amine-reactive iTRAQ or TMT^TM^ multiplex tags [221,241]. Recently, the CITe-Id (covalent inhibitor target-site identification) platform was reported [231], enabling unbiased identification and detection of modified proteins and inhibitor target site in the whole proteome by competing drug-derived desthiobiotinylated ABP with its parent inhibitor (Figure 11C). The success of this approach was illustrated with one-step ABP THZ1-DTB, a desthiobiotinylated analogue of cyclin-dependent kinase (CDK7) inhibitor THZ1, focusing on the eight cysteine residues competitively modified by THZ1 in a dose-dependent manner [231]. Among the newly identified THZ1 targets was Cys840 of PKN3, and CITe-Id streamlined the development of first-in-class PKN3 inhibitor JZ128. Proteomic platforms to assess the global electrophile selectivity are under development [242], as are improved competitive platforms to assess covalent inhibitor reactivity [225].

*Reversible inhibition and reversibility assays.* Direct chemoproteomic assessment of cellular protein targets can be challenging for reversible covalent inhibitors, as proteolytic digestion can induce dissociation of a reversible covalent ABP. Successful identification of cellular targets for (slow) reversible covalent cyanimides targeting UCHL1 has been reported with biotinylated one-step ABP 11RK73 and clickable two-step ABP 8RK64 [207]. The proteome-wide reactivity of reversible inhibitors is typically assessed in competitive proteomic experiments, which is not necessarily restricted to covalent binding modes [239,243]. The Cravatt group [244] demonstrated that it is not only possible to evaluate the proteome-wide reactivity of reversible covalent cysteine-directing compounds with competitive isoTOP-ABPP, but that adaptation of this method by the introduction of a gel filtration (GF) step before treatment with the thiol-alkylating ABP can be employed to evaluate the reversibility of the covalent adduct.

*Quantification of covalent occupancy.* Application of broad-spectrum ABPs to monitor ligand target engagement in native systems has been performed in model organisms (in vivo or ex vivo) and human tissue (ex vivo), with quantitation of relative protein abundance in presence of inhibitor compared to an untreated sample [104,198]. Occasionally, competitive ABPP experiments with drug-derived ABPs are performed. Time-dependent JAK3 occupancy in mouse spleen upon oral administration of ritlecitinib (PF-06651600) was monitored with drug-derived ABP PF-06789402: homogenized spleens were treated (ex vivo) with drug-derived ABP PF-06789402, enriched for ABP-modified proteins, and each sample was treated with a unique isobaric TMT-10plex tag to label (reactive) amines for multiplexed MS/MS analysis [199].

### 6.3. Homogeneous (Plate-Based) Platforms

Gel electrophoresis (Section 6.1) and chemoproteomic platforms (Section 6.2) require the removal of the unbound ABP or enrichment for modified proteins prior to the detection of a covalent protein–ABP adduct. In this section, we will discuss a few approaches that enable direct covalent adduct detection in a complex mixture (compatible with in situ/live cell imaging).

**Figure 12:**
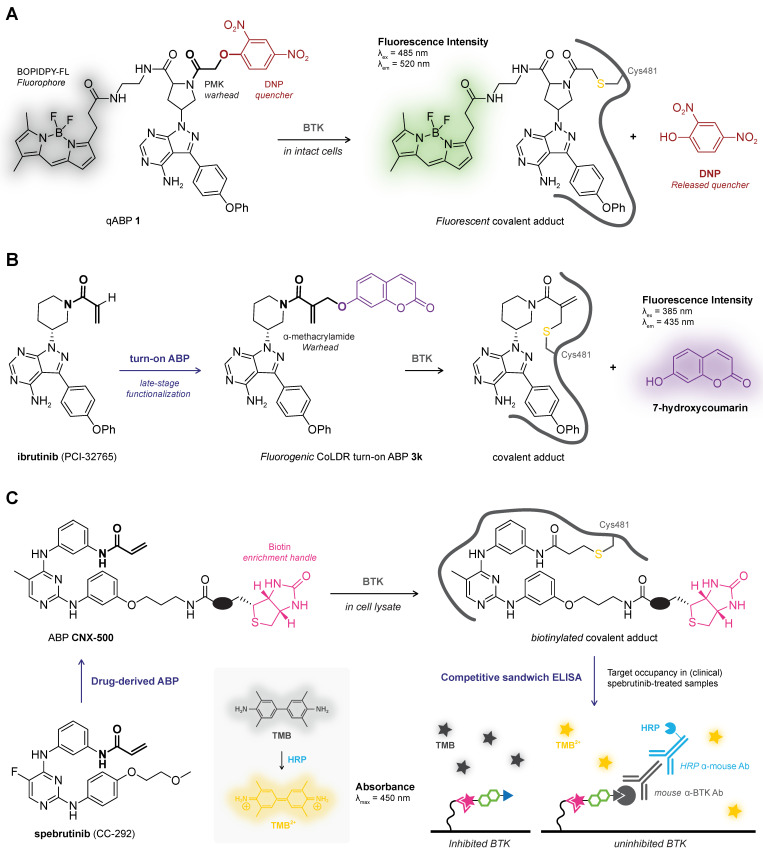
Homogeneous (plate-based) approaches to detect covalent adduct formation with drug-derived ABPs. (**A**) Quenched fluorescent ABP (qABP) with a recognition element based on the ibrutinib scaffold to selectively target BTK [252]. The DNP (2,4-dinitrophenyl) quencher is expelled upon covalent adduct formation, enabling fluorescence detection of the BODIPY-FL fluorophore in the covalent adduct but not in unbound or noncovalently bound qABP. (**B**) Covalent ligand directed release (CoLDR) chemistry to generate turn-on fluorogenic ABPs [255]. Adduct formation of ibrutinib-derived turn-on fluorogenic ABP **3k** with BTK is monitored by fluorescence intensity as thiol addition to the substituted α-methacrylamide warhead results in release of fluorescent 7-hydroxycoumarin. (**C**) Quantification of cellular BTK occupancy in a competitive ELISA (enzyme-linked immunosorbent assay) experiment [262]. Spebrutinib-treated lysates originating from tissue (culture) or clinical samples are incubated with biotinylated ABP CNX-500 to detect free, uninhibited BTK. Biotinylated BTK–ABP adducts are captured on a streptavidin-coated ELISA plate, treated with primary mouse α-BTK Ab and secondary HRP anti-mouse Ab, and developed by addition of HRP substrate tetramethyl benzidine (TMB). Uninhibited BTK is quantified from the concentration of the yellow HRP product TMB^2+^, with calculation of inhibitor occupancy from the OD_450_ in the treated samples relative to the untreated sample.

Traditional fluorescent ABPs suffer from a high fluorescence background as they are also fluorescent in their unbound state, and are thus less suitable for homogeneous applications that do not involve removal of the unbound ABP (e.g., live cell imaging, in vitro microplate-based activity assays). Turn-on fluorogenic and quenched ABPs are a subtype of fluorescent ABPs that only become fluorescent after covalent adduct formation. Quenched ABPs (qABPs) were originally developed in the Bogyo lab [245] to enable dynamic imaging of cysteine protease activity in living cells. Here, the fluorophore is ‘dark’ until the quencher is released/removed upon covalent thiol addition, generating a fluorescent covalent enzyme–inhibitor adduct. Adduct formation can be monitored by traditional in-gel fluorescence, but the low intrinsic fluorescent background also enables monitoring fluorescence intensity in homogeneous plate-based read-outs; non-invasive real-time optical imaging of cysteine protease activity is possible in intact (live) cells, and even in vivo [246,247,248]. Most qABPs targeting serines [249,250] or cysteines [182,246,247,248,251] were developed as chemical tools to study enzyme activity. Target-selective qABPs typically have a peptidic recognition element with the exception of a BTK kinase qABP derived from the noncovalent core of ibrutinib [252] (Figure 12A). The major drawback to turning a covalent ligand into a qABP is the mandatory replacement of the warhead with a suitable electrophile (e.g., acyloxymethyl ketone (AOMK) [245], phenoxymethyl ketone (PMK) [246], urea triazole [249,253]) to enable nucleophilic substitution of the quencher/fluorophore upon covalent adduct formation: the thiol-reactive electrophiles consisting of a carbonyl with a leaving group on the α-carbon may have a different intrinsic chemical reactivity than the original warhead [26,27,254], and the qABP (thiol) reactivity may no longer be representative of the parent ligand.

A more generally applicable approach for thiol-reactive covalent small molecule inhibitors was recently reported by the London group [255]: CoLDR (Covalent Ligand Directed Release) turn-on ABPs were generated by late-stage functionalization of covalent inhibitors containing the popular acrylamide warhead (Figure 12B). Modification of the acrylamide warhead on the α-carbon generates substituted α-methacrylamide warheads that release a (detectable) leaving group upon thiol addition, turning the covalent inhibitor into a turn-on fluorogenic, chemiluminescent, or otherwise functionalized ABP while maintaining the acrylamide geometry. This elegant approach was illustrated with a turn-on fluorogenic ABP based on covalent BTK inhibitor ibrutinib, which releases a fluorescent 7-hydroxycoumarin group upon thiol addition allowing homogeneous plate-based kinetic detection of irreversible covalent adduct formation. The versatility of this approach was illustrated with turn-on fluorogenic ABPs based on covalent EGFR inhibitor afatinib, covalent KRAS^G12C^ inhibitor AMG 510 functionalized with coumarin, and a chemiluminescent ibrutinib-based ABP. Note that turn-on fluorescence probes do not have to be covalent as there are examples of increased fluorescence induced by a noncovalent binding event [256], and covalent adduct formation with the desired target should be validated with orthogonal techniques such as intact protein analysis. Alternatively, two-step ABPs can be employed as bioorthogonal fluorogenic probes, reacting the protein–ABP adduct with photophysical quenched fluorogenic dyes (e.g., azido-BODIPY, dibenzocyclooctyne) that are activated by Click chemistry [197,257,258].

To this date, qABPs and turn-on ABPs have limited clinical applications as the optical signal of most fluorophores is plagued by insufficient tissue penetration, thus obstructing their application as non-invasive diagnostic tools in living patients [259]. Research endeavors in the qABP field have since progressed to advanced theranostic probes that combine detection and simultaneous inhibition of cathepsins with induction of sensitivity to photodynamic therapy (PDT) at the sites with high (aberrant) protease activity [260,261], which may one day find application in non-invasive (clinical) diagnosis and treatment.

*Quantification of covalent occupancy.* Relative fluorescence intensity can be employed to quantify covalent adduct formation with quenched or turn-on fluorogenic ABPs [255]. Recently, competitive approaches employing biotin-labeled ABPs to assess the cellular occupancy of covalent BTK inhibitors have been reported, where detection of the covalent BTK–ABP adduct is facilitated by an ELISA (enzyme-linked immunosorbent assay) [12,13,262] or an ALPHA (amplified luminescent proximity homogeneous assay) [55]. BTK occupancy is calculated from the normalization of the signal in inhibitor-treated samples to the untreated control as covalent BTK–ABP adduct is formed with uninhibited BTK but not with BTK–inhibitor adducts. Cellular BTK occupancy of irreversible covalent BTK inhibitor spebrutinib (CC-292) in human B cell lysate was assessed using spebrutinib-derived biotinylated ABP CNX-500 (Figure 12C) [262], with the capture of the biotinylated BTK–ABP adducts on streptavidin-coated ELISA plates. Uninhibited BTK was quantified from the optical density (OD_450_) originating from BTK–ABP adduct after subsequent addition of a primary BTK antibody, a secondary antibody modified with horseradish peroxidase (HRP), and development with the HRP substrate. The stepwise ELISA technology has been employed to assess BTK occupancy in the development of various covalent BTK inhibitors: zanubrutinib (BGB-3111) with zanubrutinib-derived biotinylated ABP P-1 on neutravidin-coated ELISA plates [12], and acalabrutinib (ACP-196) with acalabrutinib-derived biotinylated ABP ACP-4016 on BTK antibody-coated ELISA plates with HRP-linked streptavidin [13]. The general BTK-selective biotinylated ABP S49 was employed rather than a drug-derived ABP to quantify cellular BTK occupancy of remibrutinib (LOU064) [87]. Finally, the Taunton lab [55] reported a high-throughput method using AlphaScreen technology, based on competition with ibrutinib-derived ABP PP-biotin, that does not only enable quantification of cellular BTK occupancy in Ramos cells but also elucidates inhibitor reversibility in wash-out experiments. An indirect competitive AlphaScreen methodology has since been used in the preclinical development of reversible BTK inhibitor rilzabrutinib (PRN-1008): BTK target occupancy in Ramos B cells was assessed using a BTK-selective biotinylated ABP [49]. Importantly, these methods do not provide direct evidence on inhibitor or ABP covalency: a stringent washing step promoting dissociation of noncovalent complexes has to be introduced to discriminate between noncovalent or covalent ligands. Nevertheless, these methodologies are attractive as they are complementary proteomic approaches by using the same biotin-labeled ABPs.

## 7. Conclusions, Current Challenges, and Future Directions

Biophysical detection of a covalent adduct is an important step in covalent drug development as a(n) (ir)reversible covalent binding mode affects the SAR analysis and kinetic behavior [60]. In this work, we reviewed the available methods for direct detection of the covalent protein–drug adduct, as opposed to deduction from the decrease of the unbound drug. To ensure the detected signal originates from a covalent protein–drug adduct, covalent adduct formation is validated with at least two orthogonal methods. With a wide variety of techniques to choose from (Table 1), method selection is dictated by compatibility with (fluorescent) read-out, the available amount and purity of protein, the complexity of the reaction mixture (from purified recombinant protein to in vivo), and the desired level of information. Beyond the simple detection of a covalent adduct, some techniques can aid the identification of the targeted amino acid residue, but often protein mutagenesis is key to validating the modified amino acid residue [70,71].

This work includes the most commonly used methods but is by no means complete. Although protein crystallography remains the most informative method for the structural evaluation of covalent adducts thus far, there is a shift toward cryo-EM [263] for the structural elucidation of large protein complexes. The phenomenal progress in the past years has resulted in highly detailed structures, wherein features of <2 Å can be resolved, but structure determination of small (<50 kDa) proteins is often hindered by intrinsically noisy micrographs and low image contrast [264,265]. Currently, single-particle cryo-EM can successfully map small molecule ligands onto (large) proteins [266,267,268,269,270], making it a promising technique to also resolve covalent adducts or as an alternative for protein–drug complexes that are difficult to crystallize.

Depending on the system studied, scientists may employ a plethora of different biophysical techniques. Importantly, covalent adduct formation may require identification of the reactive metabolite to assess covalent adduct formation with prodrugs (e.g., omeprazole [271]). Other challenging systems involve (membrane-bound) protein targets that are inactive in isolation or in absence of the other components of a protein complex. The majority of the described techniques are compatible with the detection of reversible covalent adducts. The main challenge is to maintain intact protein–drug adduct during sample preparation as the sample preparation can induce target dissociation for reversible covalent ligands. The intrinsic property of a reversible covalent ligand to dissociate from its protein target upon protein denaturation [54,72] or chasing with an irreversible competitive tracer [74] has been exploited to assess the binding reversibility of various clinically approved TCIs [88,95].

The unavailability of (quantitative) high-throughput screening techniques can hamper the widespread discovery of covalent ligands. Although intact protein MS [89] can overcome this hurdle, potential hits should always be validated in a functional assay [39], as covalent modification does not necessarily mean altered protein function [71]. Another factor is monitoring and quantifying covalent target occupancy in living patients, since drug levels in serum are not representative for irreversible binders. MS-based assays to quantify target engagement have been developed [100], though practical application in clinical drug development is still limited by the optimization for each individual protein–drug pair.

Beyond validation of covalent adduct formation with the desired target, ABPP has the advantage that it can be used to evaluate target selectivity in a cellular, biological setting. Although detection of low-abundance proteins remains a challenge, the approach is in general very versatile, as evidenced by the various subtypes of ABPP. The prevalent indirect competitive ABPP with a general reactive ABP has successfully been employed to identify off-target protein targets in patient-derived samples [191], but this indirect approach is biased for the amino acid residues labeled by the general ABP and will miss labeling of other residues. Here, opportunities arise for the lower throughput use of drug-derived ABPs that have the same selectivity as the parent drug. Drug-derived ABPs have since been used to evaluate the target selectivity of approved covalent inhibitors [103,200], quantify target occupancy in inhibitor-treated patients [199,262], and identify novel targetable proteins [207]. Further developments in ABP techniques are always limited by the deviation from the parent drug when a tag/handle is introduced. At the same time, general indirect (non-drug derived) methods to evaluate proteome-wide electrophile reactivity toward other nucleophilic residues (e.g., histidine, arginine, lysine) [23,242,272,273,274,275,276] are becoming more prevalent as these nucleophilic residues are attractive targets (e.g., Arg12 in oncogenic mutant KRAS^G12R^ [277] and catalytic Lys271 in BCR-ABL [278]). All taken together, such chemoproteomic approaches will likely become an integral part of standard covalent drug development to identify covalently modified off-target proteins at an early stage [279], thereby further derisking covalent drug development [2].

To conclude, there is a broad toolbox available for the evaluation and detection of covalent protein–drug adducts, ranging from recombinant proteins to live patients. These techniques are instrumental in the evaluation of covalent drug reactivity and selectivity, and have guided covalent drug development programs and SAR optimization studies. Beyond the methods covered in this review, novel techniques will continue to be developed and improved to cater to the exciting and fast-paced field of covalent drug development.

## Figures and Tables

**Table 1:** Technologies for direct detection of covalent protein–drug adducts included in this review.

	Prerequisites		Compatibility		Structural		Characterization		
**Detection Method**	Ligand Resynthesis	Protein Optimization	Reversible Covalent	Whole proteome ^a^	Modified Amino Acid ^b^	Bond Isoform	Covalent Occupancy	Reversibility	**Notes**
2. Mass Spectrometry									Relatively fast and easy. Bottom-up MS(/MS) compatible with large proteins (in mixtures)
2.1. Top-down MS	−	+	+	~ ^c^	−	−	+	+	
2.2. Bottom-up MS	−	−	~	+	~ ^d^	−	~	−	
2.3. MS/MS	−	−	~	+	+	−	+	−	
3. Protein Crystallography	−	+	++	-	+	+	-	−	Most informative but laborious
4. Intrinsic Fluorescence/Absorbance	− ^e^	−	+	-	−	−	~	+	Limited ligand compatibility
5. Nuclear Magnetic Resonance	+	~	++	+	~	++	+	+	Compatible with labile adduct detection in solution
6. Activity-based Protein Profiling									Detection of modified (off-) target proteins in whole proteomes
6.1. Gel Electrophoresis	+	−	~	++	−	−	+	+	
6.2. Chemoproteomic Platforms	+	−	~	++	+	−	~ ^f^	+	
6.3. Homogeneous/plate-based	+	−	+	++	−	−	+	−	

^a^ Adduct formation in complex mixtures (e.g., lysates, live cells, or in vivo). ^b^ Direct detection, not including indirect identification through site-directed mutagenesis of the modified amino acid. ^c^ Requires enrichment for (modified) protein target. ^d^ Identification of the peptide containing the modified amino acid residue. ^e^ Prerequisite for drug ligand class: covalent adduct formation must induce a change in intrinsic spectroscopic properties (e.g., fluorescence, absorbance). ^f^ Typically assessed in (indirect) competition assays.

## Data Availability

Not applicable.

## Abbreviations

ABP	activity-based probe
ABPP	activity-based protein profiling
ADME	absorption, distribution, metabolism, and excretion
ALPHA	amplified luminescent proximity homogeneous assay
AOMK	acyloxymethyl ketone
APT	attached proton test
BME	β-mercaptoethanol
BODIPY	fluorinated boron-dipyrromethene
CAA	chloroacetamide
CITe-Id	covalent inhibitor target-site identification
CoLDR	covalent ligand directed release
CRI	covalent reversible inhibitor
cryo-EM	cryogenic electron microscopy
CuAAC	Huisgen copper-catalyzed alkyne–azide cycloaddition
DNP	2,4-dinitrophenyl
DPS	2,2′-dipyridyldisulfide
DTT	dithiothreitol
DUB	deubiquitinating enzyme
ELISA	enzyme-linked immunosorbent assay
FBDD	fragment-based drug development
FBLD	fragment-based ligand discovery
FDA	food and drug administration (USA)
FFPE	formalin fixed paraffin embedded
G12C-TE	KRAS^G12C^ target engagement
GSH	glutathione
GST	glutathione S-transferase
HCV	hepatitis C virus
His-	polyhistidine tag
HTS	high-throughput screening
IAc	iodoacetamide
ICC	intrahepatic cholangiocarcinoma
IEDDA	inverse electron demand Diels Alder
isoDTB	isotopically-labeled desthiobiotin
isoTOP-ABPP	isotopic tandem orthogonal activity-based protein profiling
iTRAQ	isobaric tag for relative and absolute quantitation
K_D_	dissociation constant
K_i_^*^	steady-state inhibition constant
LC	liquid chromatography
MMTS	S-methyl methanethiosulfonate
MS	mass spectrometry
NMR	nuclear magnetic resonance
NSCLC	non-small cell lung carcinoma
ODN	odanacatib
PD	pharmacodynamics
PDB	protein data bank
PDT	photodynamic therapy
PET	photo-induced electron transfer
PK	pharmacokinetics
PMK	phenoxymethyl ketone
Prg	propargyl
PROTAC	proteolysis-targeting chimera
qABP	quenched ABP
Rho	rhodamine
SAR	structure-activity relationship
SARS-CoV-2	severe acute respiratory syndrome coronavirus 2
SDS-PAGE	sodium dodecyl sulfate polyacrylamide gel electrophoresis
SILAC	stable-isotope labeling by amino acids in cell culture
SPAAC	strain-promoted alkyne–azide cycloaddition
SUMO	small ubiquitin-related modifier
TAMRA/TMR	tetramethylrhodamine
TCEP	tris(2-carboxyethyl)phosphine
TCI	targeted covalent inhibitor
TIC	total ion count
THF	tetrahydrofuran
THL	tetrahydrolipstatin
TMB	tetramethyl benzidine
TMT	tandem mass tag
Ub	ubiquitin
VME	vinyl methyl ester
XRD	X-ray diffraction

## Appendix A

**Table A1:** FDA approved drugs (2020–2022) with a known covalent mechanism of action ^a^.

**Active Ingredient ^b^**	**Drug Brand Name**	**Sponsor ^c^**	**Approval Year**	**Target**	**Indication**	**Treatment Area**	**Warhead**	**Reversibility**	**Target Residue**	**Ref**
Lurbinectedin (PM01183)	Zepzelca	Pharma Mar	2020	DNA minor groove (alkylation)	Metastatic SCLC	Cancer	Highly reactive carbinolamine (Imine intermediate)	Reversible	Guanine N2	[208]
Remdesivir (GS-5734) ^d^	Veklury	Gilead Sciences	2020	SARS-CoV-2 RdRp SARS-CoV-2 M^pro^	COVID-19	Anti-microbial	Nitrile	Reversible	Ser861 (RdRp) Cys145 (M^pro^)	[64,280]
Sotorasib (AMG 510)	Lumakras	Amgen	2021	KRAS^G12C^	KRAS^G12C^-mutated NSCLC	Cancer	Acrylamide	Irreversible	Cys12	[18]
Mobocertinib (TAK788)	Exkivity	Takeda	2021	EGFR^ex20ins^	Metastatic EGFR^ex20ins^-mutated NSCLC	Cancer	Acrylamide	Irreversible	Cys797	[19]
Nirmatrelvir (PF-07321332)	Paxlovid	Pfizer	2021 ^e^	SARS-CoV-2 M^pro^	COVID-19	Anti-microbial	Nitrile	Reversible	Cys145	[47,281]
Futibatinib (TAS-120)	Lytgobi	Taiho Oncology	2022	FGFR1-4	FGFR2 fusion-positive ICC	Cancer	Acrylamide	Irreversible	Cys488 (FGFR1) Cys492 (FGFR2-IIIb)	[282]
Adagrasib (MRTX849)	Krazati	Mirati Therapeutics	2022	KRAS^G12C^	KRAS^G12C^-mutated NSCLC	Cancer	Acrylamide	Irreversible	Cys12	[103]

Update of the FDA approved covalent drugs (1900–2019) that can be found in Table S1 of citation [20]. ^a^ Data source: FDA database. ^b^ Only the New Chemical Entity (NCE) approved in the specified year is reported for combination drugs. ^c^ Refers to the original sponsor for FDA first approval. ^d^ Covalent binding mode suspected based on covalent docking studies. ^e^ Emergency use authorization.

## References

[1] Singh J., Petter R.C., Baillie T.A., Whitty A. (2011). The resurgence of covalent drugs. Nat. Rev. Drug Discov..

[2] Johnson D.S., Weerapana E., Cravatt B.F. (2010). Strategies for discovering and derisking covalent, irreversible enzyme inhibitors. Future Med. Chem..

[3] Vane J.R., Botting R.M. (2003). The mechanism of action of aspirin. Thromb. Res..

[4] Yocum R.R., Rasmussen J.R., Strominger J.L. (1980). The mechanism of action of penicillin. Penicillin acylates the active site of Bacillus stearothermophilus D-alanine carboxypeptidase. J. Biol. Chem..

[5] Ding Z., Kim S., Dorsam R.T., Jin J., Kunapuli S.P. (2003). Inactivation of the human P2Y12 receptor by thiol reagents requires interaction with both extracellular cysteine residues, Cys17 and Cys270. Blood.

[6] Shin J.M., Cho Y.M., Sachs G. (2004). Chemistry of Covalent Inhibition of the Gastric (H+, K+)-ATPase by Proton Pump Inhibitors. J. Am. Chem. Soc..

[7] Bauer R.A. (2015). Covalent inhibitors in drug discovery: From accidental discoveries to avoided liabilities and designed therapies. Drug Discov. Today.

[8] Gehringer M. (2020). Covalent inhibitors: Back on track?. Future Med. Chem..

[9] Barf T., Kaptein A. (2012). Irreversible Protein Kinase Inhibitors: Balancing the Benefits and Risks. J. Med. Chem..

[10] Chaikuad A., Koch P., Laufer S.A., Knapp S. (2018). The Cysteinome of Protein Kinases as a Target in Drug Development. Angew. Chem. Int. Ed..

[11] Honigberg L.A., Smith A.M., Sirisawad M., Verner E., Loury D., Chang B., Li S., Pan Z., Thamm D.H., Miller R.A. (2010). The Bruton tyrosine kinase inhibitor PCI-32765 blocks B-cell activation and is efficacious in models of autoimmune disease and B-cell malignancy. Proc. Natl. Acad. Sci. USA.

[12] Guo Y., Liu Y., Hu N., Yu D., Zhou C., Shi G., Zhang B., Wei M., Liu J., Luo L. (2019). Discovery of Zanubrutinib (BGB-3111), a Novel, Potent, and Selective Covalent Inhibitor of Bruton’s Tyrosine Kinase. J. Med. Chem..

[13] Barf T., Covey T., Izumi R., van de Kar B., Gulrajani M., van Lith B., van Hoek M., de Zwart E., Mittag D., Demont D. (2017). Acalabrutinib (ACP-196): A Covalent Bruton Tyrosine Kinase Inhibitor with a Differentiated Selectivity and In Vivo Potency Profile. J. Pharmacol. Exp. Ther..

[14] Solca F., Dahl G., Zoephel A., Bader G., Sanderson M., Klein C., Kraemer O., Himmelsbach F., Haaksma E., Adolf G.R. (2012). Target Binding Properties and Cellular Activity of Afatinib (BIBW 2992), an Irreversible ErbB Family Blocker. J. Pharmacol. Exp. Ther..

[15] Cross D.A.E., Ashton S.E., Ghiorghiu S., Eberlein C., Nebhan C.A., Spitzler P.J., Orme J.P., Finlay M.R.V., Ward R.A., Mellor M.J. (2014). AZD9291, an Irreversible EGFR TKI, Overcomes T790M-Mediated Resistance to EGFR Inhibitors in Lung Cancer. Cancer Discov..

[16] Rabindran S.K., Discafani C.M., Rosfjord E.C., Baxter M., Floyd M.B., Golas J., Hallett W.A., Johnson B.D., Nilakantan R., Overbeek E. (2004). Antitumor Activity of HKI-272, an Orally Active, Irreversible Inhibitor of the HER-2 Tyrosine Kinase. Cancer Res..

[17] Gonzales A.J., Hook K.E., Althaus I.W., Ellis P.A., Trachet E., Delaney A.M., Harvey P.J., Ellis T.A., Amato D.M., Nelson J.M. (2008). Antitumor activity and pharmacokinetic properties of PF-00299804, a second-generation irreversible pan-erbB receptor tyrosine kinase inhibitor. Mol. Cancer Ther..

[18] Lanman B.A., Allen J.R., Allen J.G., Amegadzie A.K., Ashton K.S., Booker S.K., Chen J.J., Chen N., Frohn M.J., Goodman G. (2020). Discovery of a Covalent Inhibitor of KRASG12C (AMG 510) for the Treatment of Solid Tumors. J. Med. Chem..

[19] Gonzalvez F., Vincent S., Baker T.E., Gould A.E., Li S., Wardwell S.D., Nadworny S., Ning Y., Zhang S., Huang W.-S. (2021). Mobocertinib (TAK-788): A Targeted Inhibitor of *EGFR* Exon 20 Insertion Mutants in Non–Small Cell Lung Cancer. Cancer Discov..

[20] De Vita E. (2021). 10 years into the resurgence of covalent drugs. Future Med. Chem..

[21] Ward R.A., Grimster N.P. (2021). The Design of Covalent-Based Inhibitors. Annu. Rep. Med. Chem..

[22] Borsari C., Keles E., McPhail J.A., Schaefer A., Sriramaratnam R., Goch W., Schaefer T., De Pascale M., Bal W., Gstaiger M. (2022). Covalent Proximity Scanning of a Distal Cysteine to Target PI3Kα. J. Am. Chem. Soc..

[23] Gehringer M., Laufer S.A. (2019). Emerging and Re-Emerging Warheads for Targeted Covalent Inhibitors: Applications in Medicinal Chemistry and Chemical Biology. J. Med. Chem..

[24] Shindo N., Ojida A. (2021). Recent progress in covalent warheads for in vivo targeting of endogenous proteins. Bioorg. Med. Chem..

[25] Ray S., Murkin A.S. (2019). New Electrophiles and Strategies for Mechanism-Based and Targeted Covalent Inhibitor Design. Biochemistry.

[26] Martin J.S., MacKenzie C.J., Fletcher D., Gilbert I.H. (2019). Characterising covalent warhead reactivity. Bioorg. Med. Chem..

[27] Lonsdale R., Burgess J., Colclough N., Davies N.L., Lenz E.M., Orton A.L., Ward R.A. (2017). Expanding the Armory: Predicting and Tuning Covalent Warhead Reactivity. J. Chem. Inf. Model..

[28] Guan I., Williams K., Pan J., Liu X. (2021). New Cysteine Covalent Modification Strategies Enable Advancement of Proteome-wide Selectivity of Kinase Modulators. Asian J. Org. Chem..

[29] Oballa R.M., Truchon J.-F., Bayly C.I., Chauret N., Day S., Crane S., Berthelette C. (2007). A generally applicable method for assessing the electrophilicity and reactivity of diverse nitrile-containing compounds. Bioorg. Med. Chem. Lett..

[30] Péczka N., Orgován Z., Ábrányi-Balogh P., Keserű G.M. (2022). Electrophilic warheads in covalent drug discovery: An overview. Expert Opin. Drug Discov..

[31] McAulay K., Bilsland A., Bon M. (2022). Reactivity of Covalent Fragments and Their Role in Fragment Based Drug Discovery. Pharmaceuticals.

[32] De Cesco S., Kurian J., Dufresne C., Mittermaier A.K., Moitessier N. (2017). Covalent inhibitors design and discovery. Eur. J. Med. Chem..

[33] Engel J., Richters A., Getlik M., Tomassi S., Keul M., Termathe M., Lategahn J., Becker C., Mayer-Wrangowski S., Grütter C. (2015). Targeting Drug Resistance in EGFR with Covalent Inhibitors: A Structure-Based Design Approach. J. Med. Chem..

[34] Zhang T., Hatcher J.M., Teng M., Gray N.S., Kostic M. (2019). Recent Advances in Selective and Irreversible Covalent Ligand Development and Validation. Cell Chem. Biol..

[35] Chen S., Lovell S., Lee S., Fellner M., Mace P.D., Bogyo M. (2021). Identification of highly selective covalent inhibitors by phage display. Nat. Biotechnol..

[36] Dalton S.E., Campos S. (2020). Covalent Small Molecules as Enabling Platforms for Drug Discovery. ChemBioChem.

[37] Campuzano I.D.G., San Miguel T., Rowe T., Onea D., Cee V.J., Arvedson T., McCarter J.D. (2015). High-Throughput Mass Spectrometric Analysis of Covalent Protein-Inhibitor Adducts for the Discovery of Irreversible Inhibitors: A Complete Workflow. J. Biomol. Screen..

[38] Lu W., Kostic M., Zhang T., Che J., Patricelli M.P., Jones L.H., Chouchani E.T., Gray N.S. (2021). Fragment-based covalent ligand discovery. RSC Chem. Biol..

[39] Keeley A., Petri L., Ábrányi-Balogh P., Keserű G.M. (2020). Covalent fragment libraries in drug discovery. Drug Discov. Today.

[40] Resnick E., Bradley A., Gan J., Douangamath A., Krojer T., Sethi R., Geurink P.P., Aimon A., Amitai G., Bellini D. (2019). Rapid Covalent-Probe Discovery by Electrophile-Fragment Screening. J. Am. Chem. Soc..

[41] Miller R.M., Paavilainen V.O., Krishnan S., Serafimova I.M., Taunton J. (2013). Electrophilic Fragment-Based Design of Reversible Covalent Kinase Inhibitors. J. Am. Chem. Soc..

[42] Kathman S.G., Statsyuk A.V. (2016). Covalent tethering of fragments for covalent probe discovery. MedChemComm.

[43] Kathman S.G., Statsyuk A.V., Hogg P. (2019). Methodology for Identification of Cysteine-Reactive Covalent Inhibitors. Functional Disulphide Bonds: Methods and Protocols.

[44] Kumalo H.M., Bhakat S., Soliman M.E.S. (2015). Theory and Applications of Covalent Docking in Drug Discovery: Merits and Pitfalls. Molecules.

[45] Lonsdale R., Ward R.A. (2018). Structure-based design of targeted covalent inhibitors. Chem. Soc. Rev..

[46] Zaidman D., Gehrtz P., Filep M., Fearon D., Gabizon R., Douangamath A., Prilusky J., Duberstein S., Cohen G., Owen C.D. (2021). An automatic pipeline for the design of irreversible derivatives identifies a potent SARS-CoV-2 Mpro inhibitor. Cell Chem. Biol..

[47] Owen Dafydd R., Allerton Charlotte M.N., Anderson Annaliesa S., Aschenbrenner L., Avery M., Berritt S., Boras B., Cardin Rhonda D., Carlo A., Coffman Karen J. (2021). An oral SARS-CoV-2 Mpro inhibitor clinical candidate for the treatment of COVID-19. Science.

[48] Tuley A., Fast W. (2018). The Taxonomy of Covalent Inhibitors. Biochemistry.

[49] Langrish C.L., Bradshaw J.M., Francesco M.R., Owens T.D., Xing Y., Shu J., LaStant J., Bisconte A., Outerbridge C., White S.D. (2021). Preclinical Efficacy and Anti-Inflammatory Mechanisms of Action of the Bruton Tyrosine Kinase Inhibitor Rilzabrutinib for Immune-Mediated Disease. J. Immunol..

[50] Gauthier J.Y., Chauret N., Cromlish W., Desmarais S., Duong L.T., Falgueyret J.-P., Kimmel D.B., Lamontagne S., Léger S., LeRiche T. (2008). The discovery of odanacatib (MK-0822), a selective inhibitor of cathepsin K. Bioorg. Med. Chem. Lett..

[51] Groll M., Berkers C.R., Ploegh H.L., Ovaa H. (2006). Crystal Structure of the Boronic Acid-Based Proteasome Inhibitor Bortezomib in Complex with the Yeast 20S Proteasome. Structure.

[52] Perni R.B., Almquist S.J., Byrn R.A., Chandorkar G., Chaturvedi P.R., Courtney L.F., Decker C.J., Dinehart K., Gates C.A., Harbeson S.L. (2006). Preclinical Profile of VX-950, a Potent, Selective, and Orally Bioavailable Inhibitor of Hepatitis C Virus NS3-4A Serine Protease. Antimicrob. Agents Chemother..

[53] Romano K.P., Ali A., Aydin C., Soumana D., Özen A., Deveau L.M., Silver C., Cao H., Newton A., Petropoulos C.J. (2012). The Molecular Basis of Drug Resistance against Hepatitis C Virus NS3/4A Protease Inhibitors. PLoS Pathog..

[54] Serafimova I.M., Pufall M.A., Krishnan S., Duda K., Cohen M.S., Maglathlin R.L., McFarland J.M., Miller R.M., Frödin M., Taunton J. (2012). Reversible targeting of noncatalytic cysteines with chemically tuned electrophiles. Nat. Chem. Biol..

[55] Bradshaw J.M., McFarland J.M., Paavilainen V.O., Bisconte A., Tam D., Phan V.T., Romanov S., Finkle D., Shu J., Patel V. (2015). Prolonged and tunable residence time using reversible covalent kinase inhibitors. Nat. Chem. Biol..

[56] Krenske E.H., Petter R.C., Houk K.N. (2016). Kinetics and Thermodynamics of Reversible Thiol Additions to Mono- and Diactivated Michael Acceptors: Implications for the Design of Drugs That Bind Covalently to Cysteines. J. Org. Chem..

[57] Basu D., Richters A., Rauh D. (2015). Structure-based design and synthesis of covalent-reversible inhibitors to overcome drug resistance in EGFR. Bioorg. Med. Chem..

[58] Forster M., Gehringer M., Laufer S.A. (2017). Recent advances in JAK3 inhibition: Isoform selectivity by covalent cysteine targeting. Bioorg. Med. Chem. Lett..

[59] U.S. Food & Drug Administration Coronavirus (COVID-19) Update: FDA Authorizes First Oral Antiviral for Treatment of COVID-19 [News Release, 2021-12-22]. https://www.fda.gov/news-events/press-announcements/coronavirus-covid-19-update-fda-authorizes-first-oral-antiviral-treatment-covid-19.

[60] Mons E., Roet S., Kim R.Q., Mulder M.P.C. (2022). A Comprehensive Guide for Assessing Covalent Inhibition in Enzymatic Assays Illustrated with Kinetic Simulations. Curr. Protoc..

[61] Strelow J.M. (2017). A Perspective on the Kinetics of Covalent and Irreversible Inhibition. SLAS Discov..

[62] McWhirter C., Ward R.A., Grimster N.P. (2021). Chapter One Kinetic mechanisms of covalent inhibition. Annual Reports in Medicinal Chemistry.

[63] Harris C.M., Foley S.E., Goedken E.R., Michalak M., Murdock S., Wilson N.S. (2018). Merits and Pitfalls in the Characterization of Covalent Inhibitors of Bruton’s Tyrosine Kinase. SLAS Discov..

[64] Al-Khafaji K., Al-Duhaidahawi D., Taskin Tok T. (2021). Using integrated computational approaches to identify safe and rapid treatment for SARS-CoV-2. J. Biomol. Struct. Dyn..

[65] Yang X., Dilweg M.A., Osemwengie D., Burggraaff L., van der Es D., Heitman L.H., Ijzerman A.P. (2020). Design and pharmacological profile of a novel covalent partial agonist for the adenosine A1 receptor. Biochem. Pharmacol..

[66] Yang X., van Veldhoven J.P.D., Offringa J., Kuiper B.J., Lenselink E.B., Heitman L.H., van der Es D., Ijzerman A.P. (2019). Development of Covalent Ligands for G Protein-Coupled Receptors: A Case for the Human Adenosine A3 Receptor. J. Med. Chem..

[67] Weichert D., Kruse A.C., Manglik A., Hiller C., Zhang C., Hübner H., Kobilka B.K., Gmeiner P. (2014). Covalent agonists for studying G protein-coupled receptor activation. Proc. Natl. Acad. Sci. USA.

[68] Grimster N.P. (2021). Covalent PROTACs: The best of both worlds?. RSC Med. Chem..

[69] Gabizon R., Shraga A., Gehrtz P., Livnah E., Shorer Y., Gurwicz N., Avram L., Unger T., Aharoni H., Albeck S. (2020). Efficient Targeted Degradation via Reversible and Irreversible Covalent PROTACs. J. Am. Chem. Soc..

[70] Li Y.-J., Du L., Wang J., Vega R., Lee T.D., Miao Y., Aldana-Masangkay G., Samuels E.R., Li B., Ouyang S.X. (2019). Allosteric Inhibition of Ubiquitin-like Modifications by a Class of Inhibitor of SUMO-Activating Enzyme. Cell Chem. Biol..

[71] Pettinger J., Le Bihan Y.-V., Widya M., van Montfort R.L.M., Jones K., Cheeseman M.D. (2017). An Irreversible Inhibitor of HSP72 that Unexpectedly Targets Lysine-56. Angew. Chem. Int. Ed..

[72] Abdeldayem A., Raouf Y.S., Constantinescu S.N., Moriggl R., Gunning P.T. (2020). Advances in covalent kinase inhibitors. Chem. Soc. Rev..

[73] Copeland R.A., Basavapathruni A., Moyer M., Scott M.P. (2011). Impact of enzyme concentration and residence time on apparent activity recovery in jump dilution analysis. Anal. Biochem..

[74] Smith S., Keul M., Engel J., Basu D., Eppmann S., Rauh D. (2017). Characterization of Covalent-Reversible EGFR Inhibitors. ACS Omega.

[75] Tailor A., Waddington J.C., Meng X., Park B.K. (2016). Mass Spectrometric and Functional Aspects of Drug–Protein Conjugation. Chem. Res. Toxicol..

[76] Zhang Y., Fonslow B.R., Shan B., Baek M.-C., Yates J.R. (2013). Protein Analysis by Shotgun/Bottom-up Proteomics. Chem. Rev..

[77] Donnelly D.P., Rawlins C.M., DeHart C.J., Fornelli L., Schachner L.F., Lin Z., Lippens J.L., Aluri K.C., Sarin R., Chen B. (2019). Best practices and benchmarks for intact protein analysis for top-down mass spectrometry. Nat. Methods.

[78] Tokmina-Lukaszewska M., Patterson A., Berry L., Scott L., Balasubramanian N., Bothner B. (2018). The Role of Mass Spectrometry in Structural Studies of Flavin-Based Electron Bifurcating Enzymes. Front. Microbiol..

[79] Hilton G.R., Benesch J.L.P. (2012). Two decades of studying non-covalent biomolecular assemblies by means of electrospray ionization mass spectrometry. J. R. Soc. Interface.

[80] Han X., Jin M., Breuker K., McLafferty F.W. (2006). Extending Top-Down Mass Spectrometry to Proteins with Masses Greater Than 200 Kilodaltons. Science.

[81] Melby J.A., Roberts D.S., Larson E.J., Brown K.A., Bayne E.F., Jin S., Ge Y. (2021). Novel Strategies to Address the Challenges in Top-Down Proteomics. J. Am. Soc. Mass Spectrom..

[82] Mons E., Jansen I.D.C., Loboda J., van Doodewaerd B.R., Hermans J., Verdoes M., van Boeckel C.A.A., van Veelen P.A., Turk B., Turk D. (2019). The Alkyne Moiety as a Latent Electrophile in Irreversible Covalent Small Molecule Inhibitors of Cathepsin K. J. Am. Chem. Soc..

[83] Mons E., Kim R.Q., van Doodewaerd B.R., van Veelen P.A., Mulder M.P.C., Ovaa H. (2021). Exploring the Versatility of the Covalent Thiol–Alkyne Reaction with Substituted Propargyl Warheads: A Deciding Role for the Cysteine Protease. J. Am. Chem. Soc..

[84] Panyain N., Godinat A., Lanyon-Hogg T., Lachiondo-Ortega S., Will E.J., Soudy C., Mondal M., Mason K., Elkhalifa S., Smith L.M. (2020). Discovery of a Potent and Selective Covalent Inhibitor and Activity-Based Probe for the Deubiquitylating Enzyme UCHL1, with Antifibrotic Activity. J. Am. Chem. Soc..

[85] Kathman S.G., Xu Z., Statsyuk A.V. (2014). A Fragment-Based Method to Discover Irreversible Covalent Inhibitors of Cysteine Proteases. J. Med. Chem..

[86] Caldwell R.D., Qiu H., Askew B.C., Bender A.T., Brugger N., Camps M., Dhanabal M., Dutt V., Eichhorn T., Gardberg A.S. (2019). Discovery of Evobrutinib: An Oral, Potent, and Highly Selective, Covalent Bruton’s Tyrosine Kinase (BTK) Inhibitor for the Treatment of Immunological Diseases. J. Med. Chem..

[87] Angst D., Gessier F., Janser P., Vulpetti A., Wälchli R., Beerli C., Littlewood-Evans A., Dawson J., Nuesslein-Hildesheim B., Wieczorek G. (2020). Discovery of LOU064 (Remibrutinib), a Potent and Highly Selective Covalent Inhibitor of Bruton’s Tyrosine Kinase. J. Med. Chem..

[88] Liclican A., Serafini L., Xing W., Czerwieniec G., Steiner B., Wang T., Brendza K.M., Lutz J.D., Keegan K.S., Ray A.S. (2020). Biochemical characterization of tirabrutinib and other irreversible inhibitors of Bruton’s tyrosine kinase reveals differences in on and off target inhibition. Biochim. Biophys. Acta Gen. Subj..

[89] Johansson H., Isabella Tsai Y.-C., Fantom K., Chung C.-W., Kümper S., Martino L., Thomas D.A., Eberl H.C., Muelbaier M., House D. (2019). Fragment-Based Covalent Ligand Screening Enables Rapid Discovery of Inhibitors for the RBR E3 Ubiquitin Ligase HOIP. J. Am. Chem. Soc..

[90] Patricelli M.P., Janes M.R., Li L.-S., Hansen R., Peters U., Kessler L.V., Chen Y., Kucharski J.M., Feng J., Ely T. (2016). Selective Inhibition of Oncogenic KRAS Output with Small Molecules Targeting the Inactive State. Cancer Discov..

[91] Kathman S.G., Span I., Smith A.T., Xu Z., Zhan J., Rosenzweig A.C., Statsyuk A.V. (2015). A Small Molecule That Switches a Ubiquitin Ligase From a Processive to a Distributive Enzymatic Mechanism. J. Am. Chem. Soc..

[92] Dubiella C., Pinch B.J., Koikawa K., Zaidman D., Poon E., Manz T.D., Nabet B., He S., Resnick E., Rogel A. (2021). Sulfopin is a covalent inhibitor of Pin1 that blocks Myc-driven tumors in vivo. Nat. Chem. Biol..

[93] Douangamath A., Fearon D., Gehrtz P., Krojer T., Lukacik P., Owen C.D., Resnick E., Strain-Damerell C., Aimon A., Ábrányi-Balogh P. (2020). Crystallographic and electrophilic fragment screening of the SARS-CoV-2 main protease. Nat. Commun..

[94] Klein B.A., Reiz B., Robertson I.M., Irving M., Li L., Sun Y.-B., Sykes B.D. (2018). Reversible Covalent Reaction of Levosimendan with Cardiac Troponin C in Vitro and in Situ. Biochemistry.

[95] Dhillon S. (2020). Tirabrutinib: First Approval. Drugs.

[96] Hansen R., Peters U., Babbar A., Chen Y., Feng J., Janes M.R., Li L.-S., Ren P., Liu Y., Zarrinkar P.P. (2018). The reactivity-driven biochemical mechanism of covalent KRASG12C inhibitors. Nat. Struct. Mol. Biol..

[97] Li K.S., Quinn J.G., Saabye M.J., Guerrero J.F.S., Nonomiya J., Lian Q., Phung W., Izrayelit Y., Walters B.T., Gustafson A. (2022). High-Throughput Kinetic Characterization of Irreversible Covalent Inhibitors of KRASG12C by Intact Protein MS and Targeted MRM. Anal. Chem..

[98] Janes M.R., Zhang J., Li L.-S., Hansen R., Peters U., Guo X., Chen Y., Babbar A., Firdaus S.J., Darjania L. (2018). Targeting KRAS Mutant Cancers with a Covalent G12C-Specific Inhibitor. Cell.

[99] Canon J., Rex K., Saiki A.Y., Mohr C., Cooke K., Bagal D., Gaida K., Holt T., Knutson C.G., Koppada N. (2019). The clinical KRAS(G12C) inhibitor AMG 510 drives anti-tumour immunity. Nature.

[100] Hansen R., Firdaus S.J., Li S., Janes M.R., Zhang J., Liu Y., Zarrinkar P.P. (2018). An Internally Controlled Quantitative Target Occupancy Assay for Covalent Inhibitors. Sci. Rep..

[101] Kantae V., Polanski R., Lewis H.J., Haider A., Barratt D., Srinivasan B. (2022). Accelerating the Validation of Endogenous On-Target Engagement and In Cellulo Kinetic Assessment for Covalent Inhibitors of KRASG12C in Early Drug Discovery. ACS Chem. Biol..

[102] Lito P., Solomon M., Li L.-S., Hansen R., Rosen N. (2016). Allele-specific inhibitors inactivate mutant KRAS G12C by a trapping mechanism. Science.

[103] Fell J.B., Fischer J.P., Baer B.R., Blake J.F., Bouhana K., Briere D.M., Brown K.D., Burgess L.E., Burns A.C., Burkard M.R. (2020). Identification of the Clinical Development Candidate MRTX849, a Covalent KRASG12C Inhibitor for the Treatment of Cancer. J. Med. Chem..

[104] Simon G.M., Niphakis M.J., Cravatt B.F. (2013). Determining target engagement in living systems. Nat. Chem. Biol..

[105] McPherson A., Gavira J.A. (2014). Introduction to protein crystallization. Acta Crystallogr. Sect. F.

[106] Berman H., Henrick K., Nakamura H. (2003). Announcing the worldwide Protein Data Bank. Nat. Struct. Mol. Biol..

[107] Berman H.M., Westbrook J., Feng Z., Gilliland G., Bhat T.N., Weissig H., Shindyalov I.N., Bourne P.E. (2000). The Protein Data Bank. Nucleic Acids Res..

[108] Bender A.T., Gardberg A., Pereira A., Johnson T., Wu Y., Grenningloh R., Head J., Morandi F., Haselmayer P., Liu-Bujalski L. (2017). Ability of Bruton’s Tyrosine Kinase Inhibitors to Sequester Y551 and Prevent Phosphorylation Determines Potency for Inhibition of Fc Receptor but not B-Cell Receptor Signaling. Mol. Pharmacol..

[109] Engel J., Smith S., Lategahn J., Tumbrink H.L., Goebel L., Becker C., Hennes E., Keul M., Unger A., Müller H. (2017). Structure-Guided Development of Covalent and Mutant-Selective Pyrazolopyrimidines to Target T790M Drug Resistance in Epidermal Growth Factor Receptor. J. Med. Chem..

[110] Groll M., Huber R., Potts B.C.M. (2006). Crystal Structures of Salinosporamide A (NPI-0052) and B (NPI-0047) in Complex with the 20S Proteasome Reveal Important Consequences of β-Lactone Ring Opening and a Mechanism for Irreversible Binding. J. Am. Chem. Soc..

[111] Uhlenbrock N., Smith S., Weisner J., Landel I., Lindemann M., Le T.A., Hardick J., Gontla R., Scheinpflug R., Czodrowski P. (2019). Structural and chemical insights into the covalent-allosteric inhibition of the protein kinase Akt. Chem. Sci..

[112] Lockbaum G.J., Henes M., Lee J.M., Timm J., Nalivaika E.A., Thompson P.R., Kurt Yilmaz N., Schiffer C.A. (2021). Pan-3C Protease Inhibitor Rupintrivir Binds SARS-CoV-2 Main Protease in a Unique Binding Mode. Biochemistry.

[113] London N., Miller R.M., Krishnan S., Uchida K., Irwin J.J., Eidam O., Gibold L., Cimermančič P., Bonnet R., Shoichet B.K. (2014). Covalent docking of large libraries for the discovery of chemical probes. Nat. Chem. Biol..

[114] Scarpino A., Ferenczy G.G., Keserű G.M. (2018). Comparative Evaluation of Covalent Docking Tools. J. Chem. Inf. Model..

[115] Gao M., Moumbock A.F.A., Qaseem A., Xu Q., Günther S. (2022). CovPDB: A high-resolution coverage of the covalent protein–ligand interactome. Nucleic Acids Res..

[116] Shraga A., Olshvang E., Davidzohn N., Khoshkenar P., Germain N., Shurrush K., Carvalho S., Avram L., Albeck S., Unger T. (2019). Covalent Docking Identifies a Potent and Selective MKK7 Inhibitor. Cell Chem. Biol..

[117] Wan X., Yang T., Cuesta A., Pang X., Balius T.E., Irwin J.J., Shoichet B.K., Taunton J. (2020). Discovery of Lysine-Targeted eIF4E Inhibitors through Covalent Docking. J. Am. Chem. Soc..

[118] Becker D., Kaczmarska Z., Arkona C., Schulz R., Tauber C., Wolber G., Hilgenfeld R., Coll M., Rademann J. (2016). Irreversible inhibitors of the 3C protease of Coxsackie virus through templated assembly of protein-binding fragments. Nat. Commun..

[119] Serafim R.A.M., da Silva Santiago A., Schwalm M.P., Hu Z., dos Reis C.V., Takarada J.E., Mezzomo P., Massirer K.B., Kudolo M., Gerstenecker S. (2022). Development of the First Covalent Monopolar Spindle Kinase 1 (MPS1/TTK) Inhibitor. J. Med. Chem..

[120] Obaidat A., Weiss J., Wahlgren B., Manam R.R., Macherla V.R., McArthur K., Chao T.-H., Palladino M.A., Lloyd G.K., Potts B.C. (2011). Proteasome Regulator Marizomib (NPI-0052) Exhibits Prolonged Inhibition, Attenuated Efflux, and Greater Cytotoxicity than Its Reversible Analogs. J. Pharmacol. Exp. Ther..

[121] Law S., Andrault P.-M., Aguda A.H., Nguyen N.T., Kruglyak N., Brayer G.D., Brömme D. (2017). Identification of mouse cathepsin K structural elements that regulate the potency of odanacatib. Biochem. J..

[122] Forster M., Chaikuad A., Bauer S.M., Holstein J., Robers M.B., Corona C.R., Gehringer M., Pfaffenrot E., Ghoreschi K., Knapp S. (2016). Selective JAK3 Inhibitors with a Covalent Reversible Binding Mode Targeting a New Induced Fit Binding Pocket. Cell Chem. Biol..

[123] Wlodawer A., Minor W., Dauter Z., Jaskolski M. (2013). Protein crystallography for aspiring crystallographers or how to avoid pitfalls and traps in macromolecular structure determination. FEBS J..

[124] Rupp B. (2009). Biomolecular Crystallography: Principles, Practice, and Application to Structural Biology.

[125] Taylor G. (2010). Introduction to phasing. Acta Crystallogr. Sect. D.

[126] Hodel A., Kim S.-H., Brunger A.T. (1992). Model bias in macromolecular crystal structures. Acta Crystallogr. Sect. A.

[127] Ferrall-Fairbanks M.C., Kieslich C.A., Platt M.O. (2020). Reassessing enzyme kinetics: Considering protease-as-substrate interactions in proteolytic networks. Proc. Natl. Acad. Sci. USA.

[128] Lee G.M., Balouch E., Goetz D.H., Lazic A., McKerrow J.H., Craik C.S. (2012). Mapping Inhibitor Binding Modes on an Active Cysteine Protease via Nuclear Magnetic Resonance Spectroscopy. Biochemistry.

[129] Hassell A.M., An G., Bledsoe R.K., Bynum J.M., Carter H.L., Deng S.-J.J., Gampe R.T., Grisard T.E., Madauss K.P., Nolte R.T. (2007). Crystallization of protein-ligand complexes. Acta Crystallogr. Sect. D.

[130] Wienen-Schmidt B., Oebbeke M., Ngo K., Heine A., Klebe G. (2021). Two Methods, One Goal: Structural Differences between Cocrystallization and Crystal Soaking to Discover Ligand Binding Poses. ChemMedChem.

[131] Wlodawer A., Minor W., Dauter Z., Jaskolski M. (2008). Protein crystallography for non-crystallographers, or how to get the best (but not more) from published macromolecular structures. FEBS J..

[132] Martz E., Sussman J.L., Decatur W., Hodis E., Jiang Y., Prilusky J. Proteopedia: Resolution. https://proteopedia.org/wiki/index.php/Resolution.

[133] Woińska M., Grabowsky S., Dominiak P.M., Woźniak K., Jayatilaka D. (2016). Hydrogen atoms can be located accurately and precisely by x-ray crystallography. Sci. Adv..

[134] Lv Z., Yuan L., Atkison J.H., Williams K.M., Vega R., Sessions E.H., Divlianska D.B., Davies C., Chen Y., Olsen S.K. (2018). Molecular mechanism of a covalent allosteric inhibitor of SUMO E1 activating enzyme. Nat. Commun..

[135] Harshbarger W., Miller C., Diedrich C., Sacchettini J. (2015). Crystal Structure of the Human 20S Proteasome in Complex with Carfilzomib. Structure.

[136] Ekkebus R., van Kasteren S.I., Kulathu Y., Scholten A., Berlin I., Geurink P.P., de Jong A., Goerdayal S., Neefjes J., Heck A.J.R. (2013). On Terminal Alkynes That Can React with Active-Site Cysteine Nucleophiles in Proteases. J. Am. Chem. Soc..

[137] Barbosa da Silva E., Dall E., Briza P., Brandstetter H., Ferreira R.S. (2019). Cruzain structures: Apocruzain and cruzain bound to S-methyl thiomethanesulfonate and implications for drug design. Acta Crystallogr. Sect. F.

[138] Karala A.R., Ruddock L.W. (2007). Does S-Methyl Methanethiosulfonate Trap the Thiol–Disulfide State of Proteins?. Antioxid. Redox Signal..

[139] Adams J., Kauffman M. (2004). Development of the Proteasome Inhibitor Velcade™ (Bortezomib). Cancer Investig..

[140] Kabir M.L., Wang F., Clayton A.H.A. (2022). Intrinsically Fluorescent Anti-Cancer Drugs. Biology.

[141] Wilson J.N., Liu W., Brown A.S., Landgraf R. (2015). Binding-induced, turn-on fluorescence of the EGFR/ERBB kinase inhibitor, lapatinib. Org. Biomol. Chem..

[142] Klüter  S., Simard  J.R., Rode H.B., Grütter C., Pawar V., Raaijmakers H.C.A., Barf T.A., Rabiller M., van Otterlo  W.A.L., Rauh  D. (2010). Characterization of Irreversible Kinase Inhibitors by Directly Detecting Covalent Bond Formation: A Tool for Dissecting Kinase Drug Resistance. ChemBioChem.

[143] Hodge C.D., Edwards R.A., Markin C.J., McDonald D., Pulvino M., Huen M.S.Y., Zhao J., Spyracopoulos L., Hendzel M.J., Glover J.N.M. (2015). Covalent Inhibition of Ubc13 Affects Ubiquitin Signaling and Reveals Active Site Elements Important for Targeting. ACS Chem. Biol..

[144] Niu L.-Y., Chen Y.-Z., Zheng H.-R., Wu L.-Z., Tung C.-H., Yang Q.-Z. (2015). Design strategies of fluorescent probes for selective detection among biothiols. Chem. Soc. Rev..

[145] Kwan A.H., Mobli M., Gooley P.R., King G.F., Mackay J.P. (2011). Macromolecular NMR spectroscopy for the non-spectroscopist. FEBS J..

[146] Maity S., Gundampati R.K., Suresh Kumar T.K. (2019). NMR Methods to Characterize Protein-Ligand Interactions. Nat. Prod. Commun..

[147] Keiffer S., Carneiro M.G., Hollander J., Kobayashi M., Pogoryelev D., Ab E., Theisgen S., Müller G., Siegal G. (2020). NMR in target driven drug discovery: Why not?. J. Biomol. NMR.

[148] Ishima R. (2015). Protein-Inhibitor Interaction Studies Using NMR. Appl. NMR Spectrosc..

[149] Sugiki T., Furuita K., Fujiwara T., Kojima C. (2018). Current NMR Techniques for Structure-Based Drug Discovery. Molecules.

[150] Krishnan V.V., Rupp B. (2012). Macromolecular Structure Determination: Comparison of X-ray Crystallography and NMR Spectroscopy. eLS.

[151] Schirò A., Carlon A., Parigi G., Murshudov G., Calderone V., Ravera E., Luchinat C. (2020). On the complementarity of X-ray and NMR data. J. Struct. Biol. X.

[152] Bjorndahl T.C., Andrew L.C., Semenchenko V., Wishart D.S. (2007). NMR Solution Structures of the Apo and Peptide-Inhibited Human Rhinovirus 3C Protease (Serotype 14):  Structural and Dynamic Comparison. Biochemistry.

[153] Zhulenkovs D., Rudevica Z., Jaudzems K., Turks M., Leonchiks A. (2014). Discovery and structure–activity relationship studies of irreversible benzisothiazolinone-based inhibitors against Staphylococcus aureus sortase A transpeptidase. Bioorg. Med. Chem..

[154] Jaudzems K., Kurbatska V., Jēkabsons A., Bobrovs R., Rudevica Z., Leonchiks A. (2020). Targeting Bacterial Sortase A with Covalent Inhibitors: 27 New Starting Points for Structure-Based Hit-to-Lead Optimization. ACS Infect. Dis..

[155] Sastry M., Fiala R., Lipman R., Tomasz M., Patel D.J. (1995). Solution Structure of the Monoalkylated Mitomycin C–DNA Complex. J. Mol. Biol..

[156] Lin C.H., Patel D.J. (1995). Solution Structure of the Covalent Duocarmycin A-DNA Duplex Complex. J. Mol. Biol..

[157] Meyer B., Peters T. (2003). NMR Spectroscopy Techniques for Screening and Identifying Ligand Binding to Protein Receptors. Angew. Chem. Int. Ed..

[158] Furukawa A., Konuma T., Yanaka S., Sugase K. (2016). Quantitative analysis of protein–ligand interactions by NMR. Prog. Nucl. Magn. Reson. Spectrosc..

[159] Olp M.D., Sprague D.J., Goetz C.J., Kathman S.G., Wynia-Smith S.L., Shishodia S., Summers S.B., Xu Z., Statsyuk A.V., Smith B.C. (2020). Covalent-Fragment Screening of BRD4 Identifies a Ligandable Site Orthogonal to the Acetyl-Lysine Binding Sites. ACS Chem. Biol..

[160] Becker W., Bhattiprolu K.C., Gubensäk N., Zangger K. (2018). Investigating Protein–Ligand Interactions by Solution Nuclear Magnetic Resonance Spectroscopy. ChemPhysChem.

[161] Felli I.C., Pierattelli R. (2022). 13C Direct Detected NMR for Challenging Systems. Chem. Rev..

[162] Cook E.C., Usher G.A., Showalter S.A., Rhoades E. (2018). Chapter Five The Use of 13C Direct-Detect NMR to Characterize Flexible and Disordered Proteins. Methods in Enzymology.

[163] Skora L., Mestan J., Fabbro D., Jahnke W., Grzesiek S. (2013). NMR reveals the allosteric opening and closing of Abelson tyrosine kinase by ATP-site and myristoyl pocket inhibitors. Proc. Natl. Acad. Sci. USA.

[164] Metzler W.J., Yanchunas J., Weigelt C., Kish K., Klei H.E., Xie D., Zhang Y., Corbett M., Tamura J.K., He B. (2008). Involvement of DPP-IV catalytic residues in enzyme–saxagliptin complex formation. Protein Sci..

[165] Harner M.J., Frank A.O., Fesik S.W. (2013). Fragment-based drug discovery using NMR spectroscopy. J. Biomol. NMR.

[166] Nagana Gowda G.A., Pascua V., Neto F.C., Raftery D. (2022). Hydrogen–Deuterium Addition and Exchange in N-Ethylmaleimide Reaction with Glutathione Detected by NMR Spectroscopy. ACS Omega.

[167] Krishnan S., Miller R.M., Tian B., Mullins R.D., Jacobson M.P., Taunton J. (2014). Design of Reversible, Cysteine-Targeted Michael Acceptors Guided by Kinetic and Computational Analysis. J. Am. Chem. Soc..

[168] Boudreau E.A., Pelczer I., Borer P.N., Heffron G.J., LaPlante S.R. (2004). Changes in drug 13C NMR chemical shifts as a tool for monitoring interactions with DNA. Biophys. Chem..

[169] Yabe Y., Guillaume D., Rich D.H. (1988). Irreversible inhibition of papain by epoxysuccinyl peptides. Carbon-13 NMR characterization of the site of alkylation. J. Am. Chem. Soc..

[170] Transue T.R., Krahn J.M., Gabel S.A., DeRose E.F., London R.E. (2004). X-ray and NMR Characterization of Covalent Complexes of Trypsin, Borate, and Alcohols. Biochemistry.

[171] Glynn S.J., Gaffney K.J., Sainz M.A., Louie S.G., Petasis N.A. (2015). Molecular characterization of the boron adducts of the proteasome inhibitor bortezomib with epigallocatechin-3-gallate and related polyphenols. Org. Biomol. Chem..

[172] Moon J.B., Coleman R.S., Hanzlik R.P. (1986). Reversible Covalent Inhibition of Papain by a Peptide Nitrile. ^13^C NMR Evidence for a Thioimidate Ester Adduct. J. Am. Chem. Soc..

[173] Thompson S.K., Halbert S.M., Bossard M.J., Tomaszek T.A., Levy M.A., Zhao B., Smith W.W., Abdel-Meguid S.S., Janson C.A., D’Alessio K.J. (1997). Design of potent and selective human cathepsin K inhibitors that span the active site. Proc. Natl. Acad. Sci. USA.

[174] Falgueyret J.-P., Oballa R.M., Okamoto O., Wesolowski G., Aubin Y., Rydzewski R.M., Prasit P., Riendeau D., Rodan S.B., Percival M.D. (2001). Novel, Nonpeptidic Cyanamides as Potent and Reversible Inhibitors of Human Cathepsins K and L. J. Med. Chem..

[175] Sorsa T., Heikkinen S., Abbott M.B., Abusamhadneh E., Laakso T., Tilgmann C., Serimaa R., Annila A., Rosevear P.R., Drakenberg T. (2001). Binding of Levosimendan, a Calcium Sensitizer, to Cardiac Troponin C. J. Biol. Chem..

[176] Robertson I.M., Pineda-Sanabria S.E., Yan Z., Kampourakis T., Sun Y.-B., Sykes B.D., Irving M. (2016). Reversible Covalent Binding to Cardiac Troponin C by the Ca2+-Sensitizer Levosimendan. Biochemistry.

[177] Liu Y., Patricelli M.P., Cravatt B.F. (1999). Activity-based protein profiling: The serine hydrolases. Proc. Natl. Acad. Sci. USA.

[178] Cravatt B.F., Wright A.T., Kozarich J.W. (2008). Activity-Based Protein Profiling: From Enzyme Chemistry to Proteomic Chemistry. Annu. Rev. Biochem..

[179] Nomura D.K., Dix M.M., Cravatt B.F. (2010). Activity-based protein profiling for biochemical pathway discovery in cancer. Nat. Rev. Cancer.

[180] Cravatt B.F., Hsu K.-L., Weerapana E. (2019). Activity-Based Protein Profiling. Current Topics in Microbiology and Immunology.

[181] Bachovchin D.A., Cravatt B.F. (2012). The pharmacological landscape and therapeutic potential of serine hydrolases. Nat. Rev. Drug Discov..

[182] Kato D., Boatright K.M., Berger A.B., Nazif T., Blum G., Ryan C., Chehade K.A.H., Salvesen G.S., Bogyo M. (2005). Activity-based probes that target diverse cysteine protease families. Nat. Chem. Biol..

[183] Chakrabarty S., Kahler J.P., van de Plassche M.A.T., Vanhoutte R., Verhelst S.H.L. (2019). Recent Advances in Activity-Based Protein Profiling of Proteases. Curr. Top. Microbiol. Immunol..

[184] Hoch D.G., Abegg D., Adibekian A. (2018). Cysteine-reactive probes and their use in chemical proteomics. Chem. Commun..

[185] Hameed D.S., Sapmaz A., Ovaa H. (2017). How Chemical Synthesis of Ubiquitin Conjugates Helps To Understand Ubiquitin Signal Transduction. Bioconjugate Chem..

[186] Patricelli M.P., Szardenings A.K., Liyanage M., Nomanbhoy T.K., Wu M., Weissig H., Aban A., Chun D., Tanner S., Kozarich J.W. (2007). Functional Interrogation of the Kinome Using Nucleotide Acyl Phosphates. Biochemistry.

[187] Deng H., Lei Q., Wu Y., He Y., Li W. (2020). Activity-based protein profiling: Recent advances in medicinal chemistry. Eur. J. Med. Chem..

[188] Fang H., Peng B., Ong S.Y., Wu Q., Li L., Yao S.Q. (2021). Recent advances in activity-based probes (ABPs) and affinity-based probes (AfBPs) for profiling of enzymes. Chem. Sci..

[189] Backus K.M., Correia B.E., Lum K.M., Forli S., Horning B.D., González-Páez G.E., Chatterjee S., Lanning B.R., Teijaro J.R., Olson A.J. (2016). Proteome-wide covalent ligand discovery in native biological systems. Nature.

[190] Lanning B.R., Whitby L.R., Dix M.M., Douhan J., Gilbert A.M., Hett E.C., Johnson T.O., Joslyn C., Kath J.C., Niessen S. (2014). A road map to evaluate the proteome-wide selectivity of covalent kinase inhibitors. Nat. Chem. Biol..

[191] van Esbroeck A.C.M., Janssen A.P.A., Cognetta A.B., Ogasawara D., Shpak G., van der Kroeg M., Kantae V., Baggelaar M.P., de Vrij F.M.S., Deng H. (2017). Activity-based protein profiling reveals off-target proteins of the FAAH inhibitor BIA 10-2474. Science.

[192] Bird R.E., Lemmel S.A., Yu X., Zhou Q.A. (2021). Bioorthogonal Chemistry and Its Applications. Bioconjugate Chem..

[193] Rostovtsev V.V., Green L.G., Fokin V.V., Sharpless K.B. (2002). A Stepwise Huisgen Cycloaddition Process: Copper(I)-Catalyzed Regioselective “Ligation” of Azides and Terminal Alkynes. Angew. Chem. Int. Ed..

[194] Parker C.G., Pratt M.R. (2020). Click Chemistry in Proteomic Investigations. Cell.

[195] Smeenk M.L.W.J., Agramunt J., Bonger K.M. (2021). Recent developments in bioorthogonal chemistry and the orthogonality within. Curr. Opin. Chem. Biol..

[196] Agard N.J., Prescher J.A., Bertozzi C.R. (2004). A Strain-Promoted [3 + 2] Azide−Alkyne Cycloaddition for Covalent Modification of Biomolecules in Living Systems. J. Am. Chem. Soc..

[197] Oliveira B.L., Guo Z., Bernardes G.J.L. (2017). Inverse electron demand Diels–Alder reactions in chemical biology. Chem. Soc. Rev..

[198] van Rooden E.J., Florea B.I., Deng H., Baggelaar M.P., van Esbroeck A.C.M., Zhou J., Overkleeft H.S., van der Stelt M. (2018). Mapping in vivo target interaction profiles of covalent inhibitors using chemical proteomics with label-free quantification. Nat. Protoc..

[199] Xu H., Jesson M.I., Seneviratne U.I., Lin T.H., Sharif M.N., Xue L., Nguyen C., Everley R.A., Trujillo J.I., Johnson D.S. (2019). PF-06651600, a Dual JAK3/TEC Family Kinase Inhibitor. ACS Chem. Biol..

[200] Yang P.-Y., Liu K., Ngai M.H., Lear M.J., Wenk M.R., Yao S.Q. (2010). Activity-Based Proteome Profiling of Potential Cellular Targets of Orlistat An FDA-Approved Drug with Anti-Tumor Activities. J. Am. Chem. Soc..

[201] Martin J.G., Ward J.A., Feyertag F., Zhang L., Couvertier S., Guckian K., Huber K.V.M., Johnson D.S. (2021). Chemoproteomic Profiling of Covalent XPO1 Inhibitors to Assess Target Engagement and Selectivity. ChemBioChem.

[202] Lee J.H., Hou X., Kummari E., Borazjani A., Edelmann M.J., Ross M.K. (2018). Endocannabinoid hydrolases in avian HD11 macrophages identified by chemoproteomics: Inactivation by small-molecule inhibitors and pathogen-induced downregulation of their activity. Mol. Cell. Biochem..

[203] Gjonaj L., Sapmaz A., Flierman D., Janssen G.M.C., van Veelen P.A., Ovaa H. (2019). Development of a DUB-selective fluorogenic substrate. Chem. Sci..

[204] van Rooden E.J., van Esbroeck A.C.M., Baggelaar M.P., Deng H., Florea B.I., Marques A.R.A., Ottenhoff R., Boot R.G., Overkleeft H.S., Aerts J.M.F.G. (2018). Chemical Proteomic Analysis of Serine Hydrolase Activity in Niemann-Pick Type C Mouse Brain. Front. Neurosci..

[205] Li W., Blankman J.L., Cravatt B.F. (2007). A Functional Proteomic Strategy to Discover Inhibitors for Uncharacterized Hydrolases. J. Am. Chem. Soc..

[206] van der Wel T., Hilhorst R., den Dulk H., van den Hooven T., Prins N.M., Wijnakker J.A.P.M., Florea B.I., Lenselink E.B., van Westen G.J.P., Ruijtenbeek R. (2020). Chemical genetics strategy to profile kinase target engagement reveals role of FES in neutrophil phagocytosis. Nat. Commun..

[207] Kooij R., Liu S., Sapmaz A., Xin B.-T., Janssen G.M.C., van Veelen P.A., Ovaa H., Dijke P.t., Geurink P.P. (2020). Small-Molecule Activity-Based Probe for Monitoring Ubiquitin C-Terminal Hydrolase L1 (UCHL1) Activity in Live Cells and Zebrafish Embryos. J. Am. Chem. Soc..

[208] Leal J., Martínez-Díez M., García-Hernández V., Moneo V., Domingo A., Bueren-Calabuig J., Negri A., Gago F., Guillén-Navarro M., Avilés P. (2010). PM01183, a new DNA minor groove covalent binder with potent in vitro and in vivo anti-tumour activity. Br. J. Pharmacol..

[209] Jia Y., Kim R.Q., Kooij R., Ovaa H., Sapmaz A., Geurink P.P. (2022). Chemical Toolkit for PARK7: Potent, Selective, and High-Throughput. J. Med. Chem..

[210] Dana D., Pathak S.K. (2020). A Review of Small Molecule Inhibitors and Functional Probes of Human Cathepsin L. Molecules.

[211] Bogyo M., Verhelst S., Bellingard-Dubouchaud V., Toba S., Greenbaum D. (2000). Selective targeting of lysosomal cysteine proteases with radiolabeled electrophilic substrate analogs. Chem. Biol..

[212] Pellegatti M. (2012). Preclinical in vivo ADME studies in drug development: A critical review. Expert Opin. Drug Metab. Toxicol..

[213] Harding C.R., Scott I.R. (1983). Fluorography—Limitations on its use for the quantitative detection of 3H- and 14C-labeled proteins in polyacrylamide gels. Anal. Biochem..

[214] Miyahisa I., Sameshima T., Hixon M.S. (2015). Rapid Determination of the Specificity Constant of Irreversible Inhibitors (kinact/KI) by Means of an Endpoint Competition Assay. Angew. Chem. Int. Ed..

[215] Tsou H.-R., Overbeek-Klumpers E.G., Hallett W.A., Reich M.F., Floyd M.B., Johnson B.D., Michalak R.S., Nilakantan R., Discafani C., Golas J. (2005). Optimization of 6,7-Disubstituted-4-(arylamino)quinoline-3-carbonitriles as Orally Active, Irreversible Inhibitors of Human Epidermal Growth Factor Receptor-2 Kinase Activity. J. Med. Chem..

[216] Scicinski J., Oronsky B., Taylor M., Luo G., Musick T., Marini J., Adams C.M., Fitch W.L. (2012). Preclinical Evaluation of the Metabolism and Disposition of RRx-001, a Novel Investigative Anticancer Agent. Drug Metab. Dispos..

[217] McClure R.A., Williams J.D. (2018). Impact of Mass Spectrometry-Based Technologies and Strategies on Chemoproteomics as a Tool for Drug Discovery. ACS Med. Chem. Lett..

[218] Counihan J.L., Ford B., Nomura D.K. (2016). Mapping proteome-wide interactions of reactive chemicals using chemoproteomic platforms. Curr. Opin. Chem. Biol..

[219] Maurais A.J., Weerapana E. (2019). Reactive-cysteine profiling for drug discovery. Curr. Opin. Chem. Biol..

[220] Neilson K.A., Ali N.A., Muralidharan S., Mirzaei M., Mariani M., Assadourian G., Lee A., van Sluyter S.C., Haynes P.A. (2011). Less label, more free: Approaches in label-free quantitative mass spectrometry. Proteomics.

[221] Rozanova S., Barkovits K., Nikolov M., Schmidt C., Urlaub H., Marcus K., Marcus K., Eisenacher M., Sitek B. (2021). Quantitative Mass Spectrometry-Based Proteomics: An Overview. Quantitative Methods in Proteomics.

[222] Wang C., Weerapana E., Blewett M.M., Cravatt B.F. (2014). A chemoproteomic platform to quantitatively map targets of lipid-derived electrophiles. Nat. Methods.

[223] Benns H.J., Wincott C.J., Tate E.W., Child M.A. (2021). Activity- and reactivity-based proteomics: Recent technological advances and applications in drug discovery. Curr. Opin. Chem. Biol..

[224] Weerapana E., Wang C., Simon G.M., Richter F., Khare S., Dillon M.B.D., Bachovchin D.A., Mowen K., Baker D., Cravatt B.F. (2010). Quantitative reactivity profiling predicts functional cysteines in proteomes. Nature.

[225] Kuljanin M., Mitchell D.C., Schweppe D.K., Gikandi A.S., Nusinow D.P., Bulloch N.J., Vinogradova E.V., Wilson D.L., Kool E.T., Mancias J.D. (2021). Reimagining high-throughput profiling of reactive cysteines for cell-based screening of large electrophile libraries. Nat. Biotechnol..

[226] Boatner L., Palafox M., Schweppe D., Backus K. (2022). CysDB: A Human Cysteine Database based on Experimental Quantitative Chemoproteomics. ChemRxiv.

[227] Hsu J.-L., Chen S.-H. (2016). Stable isotope dimethyl labelling for quantitative proteomics and beyond. Philos. Trans. R. Soc. A.

[228] Adibekian A., Martin B.R., Wang C., Hsu K.-L., Bachovchin D.A., Niessen S., Hoover H., Cravatt B.F. (2011). Click-generated triazole ureas as ultrapotent in vivo–active serine hydrolase inhibitors. Nat. Chem. Biol..

[229] Mann M. (2006). Functional and quantitative proteomics using SILAC. Nat. Rev. Mol. Cell Biol..

[230] Ong S.-E., Blagoev B., Kratchmarova I., Kristensen D.B., Steen H., Pandey A., Mann M. (2002). Stable Isotope Labeling by Amino Acids in Cell Culture, SILAC, as a Simple and Accurate Approach to Expression Proteomics. Mol. Cell. Proteom..

[231] Browne C.M., Jiang B., Ficarro S.B., Doctor Z.M., Johnson J.L., Card J.D., Sivakumaren S.C., Alexander W.M., Yaron T.M., Murphy C.J. (2019). A Chemoproteomic Strategy for Direct and Proteome-Wide Covalent Inhibitor Target-Site Identification. J. Am. Chem. Soc..

[232] Chen X., Sun Y., Zhang T., Shu L., Roepstorff P., Yang F. (2021). Quantitative Proteomics Using Isobaric Labeling: A Practical Guide. Genom. Proteom. Bioinf..

[233] Weerapana E., Speers A.E., Cravatt B.F. (2007). Tandem orthogonal proteolysis-activity-based protein profiling (TOP-ABPP)—A general method for mapping sites of probe modification in proteomes. Nat. Protoc..

[234] Zanon P.R.A., Lewald L., Hacker S.M. (2020). Isotopically Labeled Desthiobiotin Azide (isoDTB) Tags Enable Global Profiling of the Bacterial Cysteinome. Angew. Chem. Int. Ed..

[235] Yang F., Gao J., Che J., Jia G., Wang C. (2018). A Dimethyl-Labeling-Based Strategy for Site-Specifically Quantitative Chemical Proteomics. Anal. Chem..

[236] Hsu J.-L., Huang S.-Y., Chow N.-H., Chen S.-H. (2003). Stable-Isotope Dimethyl Labeling for Quantitative Proteomics. Anal. Chem..

[237] Boersema P.J., Raijmakers R., Lemeer S., Mohammed S., Heck A.J.R. (2009). Multiplex peptide stable isotope dimethyl labeling for quantitative proteomics. Nat. Protoc..

[238] Li N., Kuo C.-L., Paniagua G., van den Elst H., Verdoes M., Willems L.I., van der Linden W.A., Ruben M., van Genderen E., Gubbens J. (2013). Relative quantification of proteasome activity by activity-based protein profiling and LC-MS/MS. Nat. Protoc..

[239] Baggelaar M.P., Chameau P.J.P., Kantae V., Hummel J., Hsu K.-L., Janssen F., van der Wel T., Soethoudt M., Deng H., den Dulk H. (2015). Highly Selective, Reversible Inhibitor Identified by Comparative Chemoproteomics Modulates Diacylglycerol Lipase Activity in Neurons. J. Am. Chem. Soc..

[240] Vinogradova E.V., Zhang X., Remillard D., Lazar D.C., Suciu R.M., Wang Y., Bianco G., Yamashita Y., Crowley V.M., Schafroth M.A. (2020). An Activity-Guided Map of Electrophile-Cysteine Interactions in Primary Human T Cells. Cell.

[241] Werner T., Becher I., Sweetman G., Doce C., Savitski M.M., Bantscheff M. (2012). High-Resolution Enabled TMT 8-plexing. Anal. Chem..

[242] Zanon P.R.A., Yu F., Musacchio P., Lewald L., Zollo M., Krauskopf K., Mrdović D., Raunft P., Maher T.E., Cigler M. (2021). Profiling the Proteome-Wide Selectivity of Diverse Electrophiles. ChemRxiv.

[243] Adibekian A., Martin B.R., Chang J.W., Hsu K.-L., Tsuboi K., Bachovchin D.A., Speers A.E., Brown S.J., Spicer T., Fernandez-Vega V. (2012). Confirming Target Engagement for Reversible Inhibitors in Vivo by Kinetically Tuned Activity-Based Probes. J. Am. Chem. Soc..

[244] Senkane K., Vinogradova E.V., Suciu R.M., Crowley V.M., Zaro B.W., Bradshaw J.M., Brameld K.A., Cravatt B.F. (2019). The Proteome-Wide Potential for Reversible Covalency at Cysteine. Angew. Chem. Int. Ed..

[245] Blum G., Mullins S.R., Keren K., Fonovič M., Jedeszko C., Rice M.J., Sloane B.F., Bogyo M. (2005). Dynamic imaging of protease activity with fluorescently quenched activity-based probes. Nat. Chem. Biol..

[246] Edgington-Mitchell L.E., Bogyo M., Verdoes M., Overkleeft H.S., Florea B.I. (2017). Live Cell Imaging and Profiling of Cysteine Cathepsin Activity Using a Quenched Activity-Based Probe. Activity-Based Proteomics: Methods and Protocols.

[247] Blum G., Weimer R.M., Edgington L.E., Adams W., Bogyo M. (2009). Comparative Assessment of Substrates and Activity Based Probes as Tools for Non-Invasive Optical Imaging of Cysteine Protease Activity. PLoS ONE.

[248] Blum G., von Degenfeld G., Merchant M.J., Blau H.M., Bogyo M. (2007). Noninvasive optical imaging of cysteine protease activity using fluorescently quenched activity-based probes. Nat. Chem. Biol..

[249] van Rooden E.J., Kohsiek M., Kreekel R., van Esbroeck A.C.M., van den Nieuwendijk A.M.C.H., Janssen A.P.A., van den Berg R.J.B.H.N., Overkleeft H.S., van der Stelt M. (2018). Design and Synthesis of Quenched Activity-based Probes for Diacylglycerol Lipase and α,β-Hydrolase Domain Containing Protein 6. Chem. Asian J..

[250] Serim S., Baer P., Verhelst S.H.L. (2015). Mixed alkyl aryl phosphonate esters as quenched fluorescent activity-based probes for serine proteases. Org. Biomol. Chem..

[251] Verdoes M., Oresic Bender K., Segal E., van der Linden W.A., Syed S., Withana N.P., Sanman L.E., Bogyo M. (2013). Improved Quenched Fluorescent Probe for Imaging of Cysteine Cathepsin Activity. J. Am. Chem. Soc..

[252] Zhang Q., Liu H., Pan Z. (2014). A general approach for the development of fluorogenic probes suitable for no-wash imaging of kinases in live cells. Chem. Commun..

[253] Janssen A.P.A., van Hengst J.M.A., Béquignon O.J.M., Deng H., van Westen G.J.P., van der Stelt M. (2019). Structure Kinetics Relationships and Molecular Dynamics Show Crucial Role for Heterocycle Leaving Group in Irreversible Diacylglycerol Lipase Inhibitors. J. Med. Chem..

[254] Petri L., Ábrányi-Balogh P., Varga P.R., Imre T., Keserű G.M. (2020). Comparative reactivity analysis of small-molecule thiol surrogates. Bioorg. Med. Chem..

[255] Reddi R.N., Resnick E., Rogel A., Rao B.V., Gabizon R., Goldenberg K., Gurwicz N., Zaidman D., Plotnikov A., Barr H. (2021). Tunable Methacrylamides for Covalent Ligand Directed Release Chemistry. J. Am. Chem. Soc..

[256] Kawahata W., Asami T., Fujii I., Sawa M. (2015). ‘Turn On/Off’ fluorescence probe for the screening of unactivated Bruton’s tyrosine kinase. Bioorg. Med. Chem. Lett..

[257] Li X., Gao X., Shi W., Ma H. (2014). Design Strategies for Water-Soluble Small Molecular Chromogenic and Fluorogenic Probes. Chem. Rev..

[258] Shie J.-J., Liu Y.-C., Lee Y.-M., Lim C., Fang J.-M., Wong C.-H. (2014). An Azido-BODIPY Probe for Glycosylation: Initiation of Strong Fluorescence upon Triazole Formation. J. Am. Chem. Soc..

[259] Blau R., Epshtein Y., Pisarevsky E., Tiram G., Israeli Dangoor S., Yeini E., Krivitsky A., Eldar-Boock A., Ben-Shushan D., Gibori H. (2018). Image-guided surgery using near-infrared Turn-ON fluorescent nanoprobes for precise detection of tumor margins. Theranostics.

[260] Ben-Nun Y., Merquiol E., Brandis A., Turk B., Scherz A., Blum G. (2015). Photodynamic Quenched Cathepsin Activity Based Probes for Cancer Detection and Macrophage Targeted Therapy. Theranostics.

[261] Weiss-Sadan T., Ben-Nun Y., Maimoun D., Merquiol E., Abd-Elrahman I., Gotsman I., Blum G. (2019). A Theranostic Cathepsin Activity-Based Probe for Noninvasive Intervention in Cardiovascular Diseases. Theranostics.

[262] Evans E.K., Tester R., Aslanian S., Karp R., Sheets M., Labenski M.T., Witowski S.R., Lounsbury H., Chaturvedi P., Mazdiyasni H. (2013). Inhibition of Btk with CC-292 Provides Early Pharmacodynamic Assessment of Activity in Mice and Humans. J. Pharmacol. Exp. Ther..

[263] Callaway E. (2020). Revolutionary cryo-EM is taking over structural biology. Nature.

[264] Chiu Y.-H., Ko K.-T., Yang T.-J., Wu K.-P., Ho M.-R., Draczkowski P., Hsu S.-T.D. (2021). Direct Visualization of a 26 kDa Protein by Cryo-Electron Microscopy Aided by a Small Scaffold Protein. Biochemistry.

[265] Liu Y., Huynh D.T., Yeates T.O. (2019). A 3.8 Å resolution cryo-EM structure of a small protein bound to an imaging scaffold. Nat. Commun..

[266] Borgnia M.J., Banerjee S., Merk A., Matthies D., Bartesaghi A., Rao P., Pierson J., Earl L.A., Falconieri V., Subramaniam S. (2016). Using Cryo-EM to Map Small Ligands on Dynamic Metabolic Enzymes: Studies with Glutamate Dehydrogenase. Mol. Pharmacol..

[267] Toelzer C., Gupta K., Yadav S.K.N., Borucu U., Davidson A.D., Kavanagh Williamson M., Shoemark D.K., Garzoni F., Staufer O., Milligan R. (2020). Free fatty acid binding pocket in the locked structure of SARS-CoV-2 spike protein. Science.

[268] Greber B.J., Perez-Bertoldi J.M., Lim K., Iavarone A.T., Toso D.B., Nogales E. (2020). The cryoelectron microscopy structure of the human CDK-activating kinase. Proc. Natl. Acad. Sci. USA.

[269] Robertson M.J., van Zundert G.C.P., Borrelli K., Skiniotis G. (2020). GemSpot: A Pipeline for Robust Modeling of Ligands into Cryo-EM Maps. Structure.

[270] Chengalroyen M.D., Mason M.K., Borsellini A., Tassoni R., Abrahams G.L., Lynch S., Ahn Y.-M., Ambler J., Young K., Crowley B.M. (2022). DNA-Dependent Binding of Nargenicin to DnaE1 Inhibits Replication in Mycobacterium tuberculosis. ACS Infect. Dis..

[271] Lindberg P., Nordberg P., Alminger T., Braendstroem A., Wallmark B. (1986). The mechanism of action of the antisecretory agent omeprazole. J. Med. Chem..

[272] Mukherjee H., Grimster N.P. (2018). Beyond cysteine: Recent developments in the area of targeted covalent inhibition. Curr. Opin. Chem. Biol..

[273] Jones L.H., Ward R.A., Grimster N.P. (2021). Chapter Four Design of next-generation covalent inhibitors: Targeting residues beyond cysteine. Annual Reports in Medicinal Chemistry.

[274] Gambini L., Baggio C., Udompholkul P., Jossart J., Salem A.F., Perry J.J.P., Pellecchia M. (2019). Covalent Inhibitors of Protein–Protein Interactions Targeting Lysine, Tyrosine, or Histidine Residues. J. Med. Chem..

[275] Pettinger J., Jones K., Cheeseman M.D. (2017). Lysine-Targeting Covalent Inhibitors. Angew. Chem. Int. Ed..

[276] Zhang Z., Guiley K.Z., Shokat K.M. (2022). Chemical acylation of an acquired serine suppresses oncogenic signaling of K-Ras(G12S). Nat. Chem. Biol..

[277] Zhang Z., Morstein J., Ecker A.K., Guiley K.Z., Shokat K.M. (2022). Chemoselective Covalent Modification of K-Ras(G12R) with a Small Molecule Electrophile. J. Am. Chem. Soc..

[278] Chen P., Sun J., Zhu C., Tang G., Wang W., Xu M., Xiang M., Zhang C.-J., Zhang Z.-M., Gao L. (2022). Cell-Active, Reversible, and Irreversible Covalent Inhibitors That Selectively Target the Catalytic Lysine of BCR-ABL Kinase. Angew. Chem. Int. Ed..

[279] Lill J.R., Mathews W.R., Rose C.M., Schirle M. (2021). Proteomics in the pharmaceutical and biotechnology industry: A look to the next decade. Expert Rev. Proteom..

[280] Aranda J., Orozco M. (2020). RNA-Dependent RNA Polymerase From SARS-CoV-2. Mechanism Of Reaction And Inhibition By Remdesivir. bioRxiv.

[281] Zhao Y., Fang C., Zhang Q., Zhang R., Zhao X., Duan Y., Wang H., Zhu Y., Feng L., Zhao J. (2022). Crystal structure of SARS-CoV-2 main protease in complex with protease inhibitor PF-07321332. Protein Cell.

[282] Kalyukina M., Yosaatmadja Y., Middleditch M.J., Patterson A.V., Smaill J.B., Squire C.J. (2019). TAS-120 Cancer Target Binding: Defining Reactivity and Revealing the First Fibroblast Growth Factor Receptor 1 (FGFR1) Irreversible Structure. ChemMedChem.

